# Exploring the postpartum return to sport and performance in Canadian elite athletes

**DOI:** 10.3389/fspor.2025.1665212

**Published:** 2025-10-07

**Authors:** Chloe M. Hewitt, M. Karen Campbell, Yun-Hee Choi, Jane S. Thornton

**Affiliations:** ^1^ Department of Epidemiology and Biostatistics, Schulich School of Medicine & Dentistry, Western University, London, ON, Canada; ^2^ Department of Paediatrics, Schulich School of Medicine & Dentistry, Western University, London, ON, Canada; ^3^ Department of Obstetrics & Gynaecology, Schulich School of Medicine & Dentistry, Western University, London, ON, Canada; ^4^ Division of Health and Therapeutics, Children’s Health Research Institute, Lawson Health Research Institute, London, ON, Canada; ^5^ Centre for Studies in Family Medicine, Schulich School of Medicine & Dentistry, Western University, London, ON, Canada; ^6^ School of Kinesiology, Western University, London, ON, Canada; ^7^ Health, Medicine and Science Department, International Olympic Committee, Lausanne, Switzerland

**Keywords:** pregnancy, postpartum, athletes, performance, competition, return

## Abstract

**Background:**

Athlete-mothers in elite sport were viewed as anomalies until very recently. Perhaps as a consequence of limited research, support and resources available for pregnant and postpartum athletes may be inadequate.

**Objective:**

To explore the experiences of athletes returning to sport and performance postpartum.

**Methods:**

Ten elite Canadian athletes who became pregnant during their sporting career and attempted to return to competitive sport after childbirth completed an online questionnaire.

**Results:**

Most participants reported that timing of conception was planned around competition schedule. During pregnancy, most athletes trained through pregnancy, but found the advice they received unsatisfactory. Lack of support for childcare within sport and balancing breastfeeding with training were commonly identified challenges. Nine of the 10 athletes reported resuming training after childbirth and five returned to competitive sport, of which four reported improved performance.

**Conclusion:**

Elite athletes in our sample were able to return to a high level of sport after childbirth, however many expressed the need for improved support through pregnancy and the postpartum period. This exploratory study reveals promising findings of how experiences are improving for pregnant and postpartum elite athletes. This sets the stage for more research to promote sport participation by athlete mothers.

## 1 Introduction

Until recent years, motherhood and participation in elite sport were generally mutually exclusive. The majority of athletes retired from elite sport upon planning to start a family (1). Today, athlete-mothers are more common, however many athletes express challenges in navigating sport and pregnancy and returning to performance postpartum due to the lack of support and evidence-based guidance (2–8).

Pregnancy and the postpartum period introduce significant mental and physical changes that require careful consideration when continuing or resuming training (9–14). While recommendations for returning to exercise or sport postpartum exist, they are primarily tailored towards recreational athletes (9, 15, 16). Furthermore, these guidelines may lack evidence-based research and therefore often are based on low-quality evidence (17, 18). This inadequate guidance for athletes recovering from the physical changes of pregnancy and childbirth, possibly for the first time, may contribute to injuries which have been observed among postpartum athletes in previous studies (19–22).

Previous research has also highlighted challenges with financial support during pregnancy and the postpartum period, including a lack of support for childcare among sport organizations (2, 4, 5, 7, 16). This lack of support can be a source of distress (10), and may cause athletes to rush into their postpartum return to training (2, 3, 9, 23, 24), further risking injury.

For new athlete-mothers, parenthood often involves a mindset shift from being solely focussed on their sport to balancing sport and caring for their child (7, 25, 26). Some athletes have struggled with this new identity (4), while it provided a new motivation for others (7, 25–27).

The media has highlighted several success stories of athlete-mothers (1, 28–30), but the stories of those who fail to return to sport postpartum may go untold. Additionally, athletes who return to sport following pregnancy are often portrayed as anomalies, which suggests that despite advances, elite sport may still not be the most welcoming environment for athlete-mothers.

By exploring factors that contribute to returning to performance postpartum, future research in these areas may be addressed. The acquisition of further information may expose the need for change in policies and guidelines concerning pregnant and postpartum elite athletes. The expected impact of informing these policies and guidelines is to facilitate the return to sport and performance for future postpartum athletes, and empower women in sport.

**Objective:** The primary objective of this study was to explore biopsychosocial factors associated with return to sport and performance postpartum among Canadian athlete-mothers, and to inform future research.

## 2 Methods

### 2.1 Study design and population

Using a cross-sectional design, an anonymized online self-report questionnaire was designed using Qualtrics (Qualtrics, 2005, Copyright 2025, Provo, Utah, USA; versions: November 2023–July 2024; available at https://www.qualtrics.com). The full questionnaire can be found in Appendix A. The questionnaire was distributed via email to potential participants. Eligible participants were Canadian female athletes participating at the national level or higher in a sport funded by Sport Canada. Inclusion criteria also required that athletes had become pregnant during their sporting career and had attempted to return to competition after childbirth. Athletes who retired prior to or upon pregnancy or did not attempt to return to competition after childbirth were excluded from the study. The eligibility criteria are presented in Table 1. The criteria were presented in the study description and confirmation of eligibility was established using a set of screening questions on Qualtrics prior to proceeding with the survey questions. The questionnaire ended for respondents who answered “no” to any of the screening questions. Participants who produced Qualtrics results were presumed to meet the criterion of having access to a computer or mobile device.

**Table 1:** Study inclusion and exclusion criteria.

Inclusion criteria	Exclusion criteria
• Female Canadian athletes who participate(d) in a sport funded by Sport Canada • Have met the minimum requirement to compete at the national level in their sport • Became pregnant during their sporting career and attempted to return to competition after childbirth • English speaking • Access to a computer or mobile device with a working keyboard and internet connection	• Athletes who did not attempt to return to competition after childbirth • Athletes who retired prior to or upon pregnancy

### 2.2 Questionnaire development

The questionnaire adapted relevant questions from *Hang up your cleats and hope for the best? A cross-sectional study investigating retired elite female rugby players' health* by Thornton et al. (31) regarding health outcomes during pregnancy and the postpartum period. Experts in sports medicine and former high-level athletes assisted with piloting and provided feedback regarding survey flow and appearance, as well as content and response options. Due to the anticipated small sample size and exploratory nature of the study, the questionnaire did not undergo a validation process or include questions from validated questionnaires.

In addition to the above, common topics which emerged from a literature review were included in order to assess perceptions of athletes in the current sample. Topics included pregnancy planning, mental health implications, sport funding, childcare options, and sociocultural attitudes.

Knowledge gaps identified from the review were addressed by developing a thorough series of quantitative and qualitative questions on these gaps. These questions primarily pertained to training and performance data and explored specific timelines, volumes and schedules of training and competition, as well as the use of guidelines for training. Another area of new exploration included the experiences of return to sport and performance following subsequent pregnancies.

### 2.3 Study variables

The questionnaire consisted of a minimum of ten sections, with the possibility of subsequent sections depending on the participant's responses. Each of the ten main sections pertained to a different topic, described in Table 2 below. The response types included multiple choice, numeric response, and open-ended text response.

**Table 2:** List of questionnaire sections based on participant characteristics.

Sections for all participants	Sections for participants who returned to sport and performance postpartum
• Demographic information • Pregnancy planning • Pregnancy experiences • Breastfeeding • Injuries and pain • Training patterns • Psychological impacts • Financial support and sport policies • Sociocultural influences	• Postpartum performance • Reflections

Participants with more than one child had an additional section for each subsequent birth. These sections addressed questions from previous sections, serving to identify any changes in experience between subsequent pregnancies.

### 2.4 Recruitment and data collection

Since no databases with contact information for Canadian athletes were accessible, a poster with a link to the survey was distributed via social media by the study coordinator and made shareable by anyone to reach as large an audience as possible. Additionally, personal connections in the sporting community through the study coordinator and investigators were used to directly contact potential participants when their contact information was publicly available. To avoid obtaining the identities and contact information of potential participants, we asked consented participants to share information about the study with other potential participants, who were able to contact the study coordinator if they wished for more information. Athletes who expressed interest in participating in the study received a follow-up email as a reminder to complete the questionnaire. The questionnaire was open between November 23, 2023 and July 24, 2024, and participants had this duration to complete the questionnaire.

### 2.5 Data analysis

A small sample size was anticipated due to the relatively small population of elite athlete-mothers. The questionnaire was widely distributed with the aim of describing experiences of athletes representing different sports and ranges of outcomes of returning to sport or performance postpartum.

The data from Qualtrics was exported and each individual question was assessed by a single analyst (CH) using SAS software V.9.4 (Cary, North Carolina, SAS Institute). Continuous variables were described using median, range and interquartile range (IQR), and categorical data were described with frequencies. Description of qualitative responses included narrative reports due to the small sample size. After analyzing in SAS, results were tabulated in Microsoft Excel (Redmond, Washington, Microsoft Corporation). Data were stored as per Western University's guidelines. The study was approved by the University of Western Ontario's Health Sciences Research Ethics Board (HSREB ID# 124169).

## 3 Results

Seventeen respondents were screened for eligibility. Three failed the eligibility criteria; one athlete did not compete at the national level and two did not become pregnant during their athletic career and give birth. Fourteen participants passed the screening and consented to participate; however, 10 completed the questionnaire. Reasons for the four incomplete surveys are unknown. Although measures were taken to prevent fraudulent responses, it is possible that these four were bot responses.

### 3.1 Demographic characteristics and sport background

Nine athletes completed the demographic characteristics section. Due to the small sample size of our study, the athlete with missing demographic data was included in the analysis of subsequent sections. Since elite Canadian athlete-mothers consist of a relatively small population, it is possible that this athlete chose not to complete the demographic and sport questions to limit the risk of identification.

The median age of respondents was 38 years (range = 29–43 years, IQR = 8.5). All participants were legally married and not separated, identified as women, and were white. Seven athletes participated in Athletics (track and field), and two were basketball players. Provincial affiliations of athletes were Alberta (*n* = 1), British Columbia (*n* = 6), New Brunswick (*n* = 1), and Quebec (*n* = 1). Athletes had participated in their sport for a median of 24 (range = 2–30) years in total and 16 (range = 2–25) years at the elite level, with two of the participants being retired from sport. All athletes reported to have represented a national team, with six as Olympic Games or World Championships as their highest level of competition.

### 3.2 Pregnancy planning

Ten participants completed the pregnancy planning section. All athletes planned their pregnancies, with six (60%) reporting that the timed conception around competition schedules, such as at the end of a season or between major competition cycles (e.g., Olympics or World Championships). One athlete initially planned to time their pregnancy around their competition schedule; however, after two years of failing to conceive she changed plans and prioritized becoming pregnant over her athletic career. One athlete described reducing her training load to optimize fertility. Another athlete described that they timed her pregnancy with her transition between sporting disciplines. Of the athletes who did not time their pregnancies around competitions, one described the lack of competitions during the COVID-19 pandemic as having an influence on starting a family.

The time required to conceive ranged from less than one month to over 12 months, with five athletes requiring over 12 months. Over half of the athletes described feeling external pressure to conceive within a certain timeframe, with the majority of pressure as a result of not wanting to miss major competitions. Of the athletes whose external pressure was from competition schedules, one also described feeling pressure to not lose funding. Another athlete felt pressure from delaying starting a family, and one felt a time constraint to conceive as a consequence of their partner also being a professional athlete.

### 3.3 Pregnancy experiences

The proportion of athletes who were first-time parents compared to parents of multiple children was equal; five (50%) athletes had one child, four (40%) had two children and one (10%) athlete had three children. All of the births were singletons. The median age of first-time mothers was 32.5 (range = 26–36) years. Only one (10%) athlete reported suffering nausea and/or vomiting while pregnant; no other complications were reported.

Two (20%) athletes reported undergoing a Caesarean section while the remainder had a vaginal delivery, and two (20%) athletes underwent episiotomies. One (10%) athlete reported enduring childbirth complications including prolonged labour and a second-degree perineal tear.

Four (40%) athletes reported postpartum complications, including an irregular menstrual cycle, nutrient deficiency, stress/urge incontinence, rectus abdominis diastasis, vaginal heaviness/pressure, and postpartum hemorrhage.

### 3.4 Breastfeeding

Ten participants reported to have breastfed. Of the nine participants who provided details on the duration of breastfeeding, seven (77%) reported breastfeeding for twelve months or longer. The other two athletes reported breastfeeding for five and six months. All (*n* = 10) athletes reported they were able to train while breastfeeding, with most (*n* = 8, 80%) reporting incorporating breastfeeding into their training schedule as somewhat difficult. When asked how breastfeeding impacted their training, athletes frequently described the unpredictability of their infants' feeding schedule as limiting their ability to train consistently. Several athletes also experienced challenges intaking enough calories to maintain adequate weight and energy levels while breastfeeding. Some athletes reported feeling exhausted while breastfeeding, with one athlete reporting having to stop breastfeeding to be able to resume training.

Of seven athletes who returned to competition after childbirth, six (86%) athletes were still breastfeeding when they first returned to competition. Most of these athletes described experiencing difficulty bringing their child to competitions. Lack of supports in place in sport were some reasons for these difficulties, including not allowing strollers or having lactation rooms at competition venues, or insufficient funding to bring their child and a caregiver for overseas competitions. Other reasons included finding someone to look after their child while competing and feeling awkward stepping away from competition as a high-level athlete to breastfeed. One athlete described sending multiple correspondences to allow their breastfeeding child to accompany them to an international competition, and eventually had to turn to the media to resolve the issue.

### 3.5 Injury/pain

Three (30%) athletes sustained an injury or musculoskeletal pain while pregnant. One athlete experienced sciatica near the end of their pregnancy, and another suffered nearly debilitating symphysis pubis dysfunction. The third athlete experienced exacerbation of an existing partial Achilles tear and ankle sprain as a result of weight gain during pregnancy.

**Figure 1:**
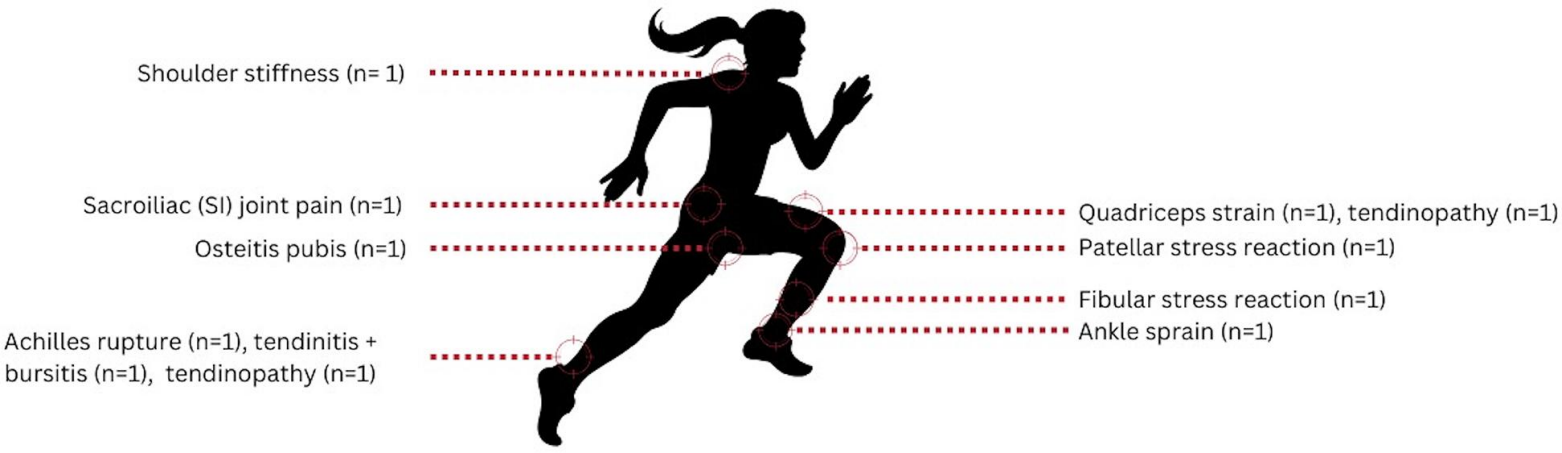
Locations and types of injuries and/or musculoskeletal pain experienced by eight participants in the postpartum period.

Eight (80%) athletes sustained an injury or musculoskeletal pain during the postpartum period. Figure 1 provides a representation of the types and locations of concerns for visualisation purposes. The Achilles and quadriceps tendinopathies were experienced by the same athlete. One athlete experienced two fibular stress reactions as well as a patellar stress reaction. The athlete who reported an Achilles tear and an ankle sprain during pregnancy had persisting symptoms postpartum. Most of the injuries occurred during sport participation postpartum, however one athlete reported shoulder tightness due to carrying a heavy child, and later reported numbness in one of her legs. Of the aforementioned injuries, only two were recurring issues prior to pregnancy, both of which pertained to the Achilles tendon.

### 3.6 Training patterns

#### 3.6.1 Training during pregnancy

Seven of the ten (70%) athletes trained while pregnant. One athlete described remaining active through their pregnancy but not doing sport-specific training, and another athlete described a lack of coaching guidance as their reason for not training while pregnant. The third athlete reporting having a low-sitting baby and was at risk for preterm labour.

Six of the seven (86%) athletes trained during all three trimesters. One athlete only trained during the second due to fatigue in the first and third trimesters but remained active. Participants trained from 24 to 40 weeks into their pregnancy, with a median of 36 weeks.

**Figure 2:**
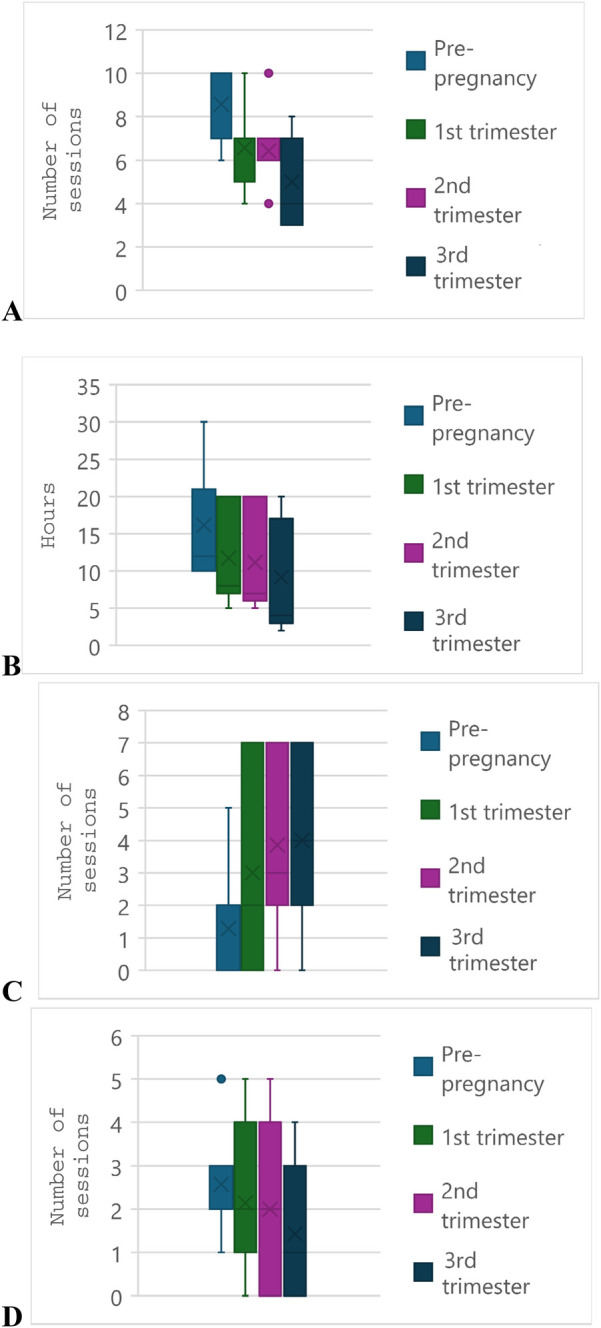
Training volumes of seven participants across trimesters of pregnancy and prior to pregnancy. **(A)** Total number of training sessions per week. **(B)** Total hours of training per week. **(C)** Number of cross-training sessions per week. **(D)** Number of strength training sessions per week. The “x” denotes the mean and the solid line through the box denotes the median. The whiskers denote the first and third quartiles, and individual data points denote outliers.

The training data of participants during pregnancy is presented in Figure 2. Overall, the number of weekly training sessions and total weekly hours of training decreased from pre-pregnancy to the first trimester among athletes. Athletes partook in a median of 10 (IQR = 3) sessions totalling 12 (IQR = 11) hours per week pre-pregnancy; during the first trimester these numbers dropped to seven (IQR = 2) and eight (IQR = 13), respectively. There was minimal change between first and second trimesters, but a further decrease in frequency and volume in the third trimester. From the second to third trimester, the median weekly number of training sessions decreased from six to four, and the median total hours decreased from seven to four. While most athletes did not partake in cross-training prior to pregnancy, the number of cross-training sessions increased as pregnancy progressed, with a median of four sessions per week by the third trimester. The number of strength training sessions per week remained the same as pre-pregnancy among most athletes, except for a decrease in the final trimester.

**Figure 3:**
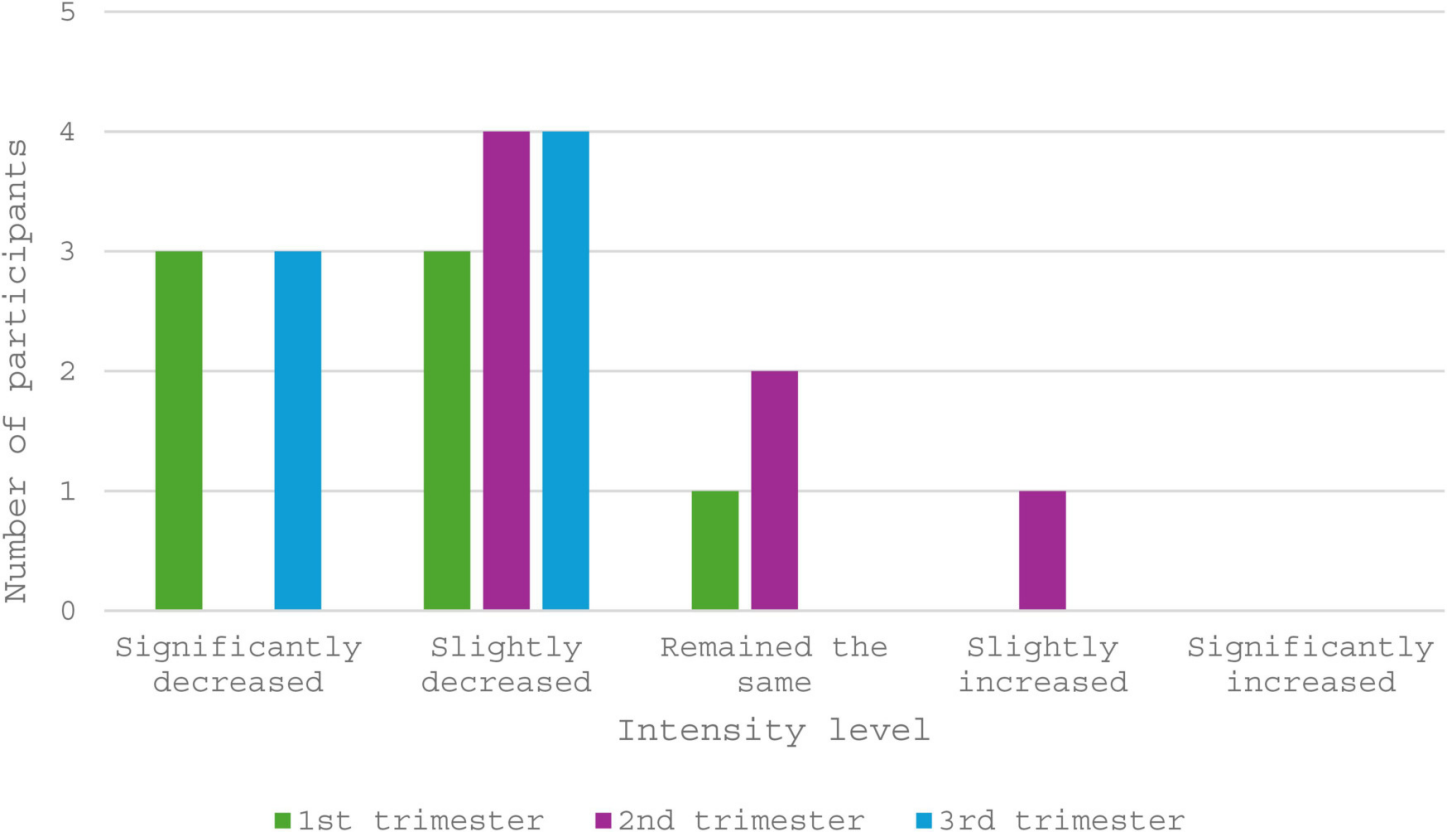
Training intensities of participants (*N* = 7) when compared to pre-pregnancy and previous trimesters.

The majority of those who trained during pregnancy (6 of 7, 86%) decreased their training intensity in the first trimester, as shown in Figure 3. Similar to the findings of training volume, intensity in the second trimester only slightly decreased for most (*n* = 4) athletes, two trained at the same intensity and one increased their intensity with respect to the first trimester. All athletes decreased their training intensity in the third trimester.

Four (57%) of the seven athletes reported following guidelines for training during pregnancy. The recommendations they received were to keep their heart rate below a certain level (150 beats per minute for one athlete, below 70% of maximum heart rate for another athlete) or to listen to their body. None of the participants felt that the guidelines were appropriate for their level of sport. Two athletes described the lack of evidence-based data available as a limitation of training recommendations, while another athlete felt that the guidelines are out-dated and geared more towards individuals who were sedentary prior to pregnancy. Similarly, one athlete felt as though the recommendations were too conservative for athletes accustomed to training at a high-intensity level.

#### 3.6.2 Training after pregnancy

Nine (90%) athletes resumed training after childbirth. One athlete had a pre-pregnancy injury that was neglected during pregnancy and persisted postpartum, preventing their return to training. The median of the first training session was five weeks postpartum (range: 2–8), with four athletes starting with cross-training as opposed to sport-specific training. The two athletes who had Caesarean deliveries returned at five and six weeks postpartum, suggesting no difference in timing of return from athletes with vaginal deliveries. The first high-intensity training session postpartum varied among athletes. One (11%) athlete had their first high-intensity session less than six weeks into postpartum training. For most (*n* = 5, 56%) athletes, this timeline was between six and 12 weeks, however 3 (33%) athletes had their first high-intensity session beyond twelve weeks of training.

**Figure 4:**
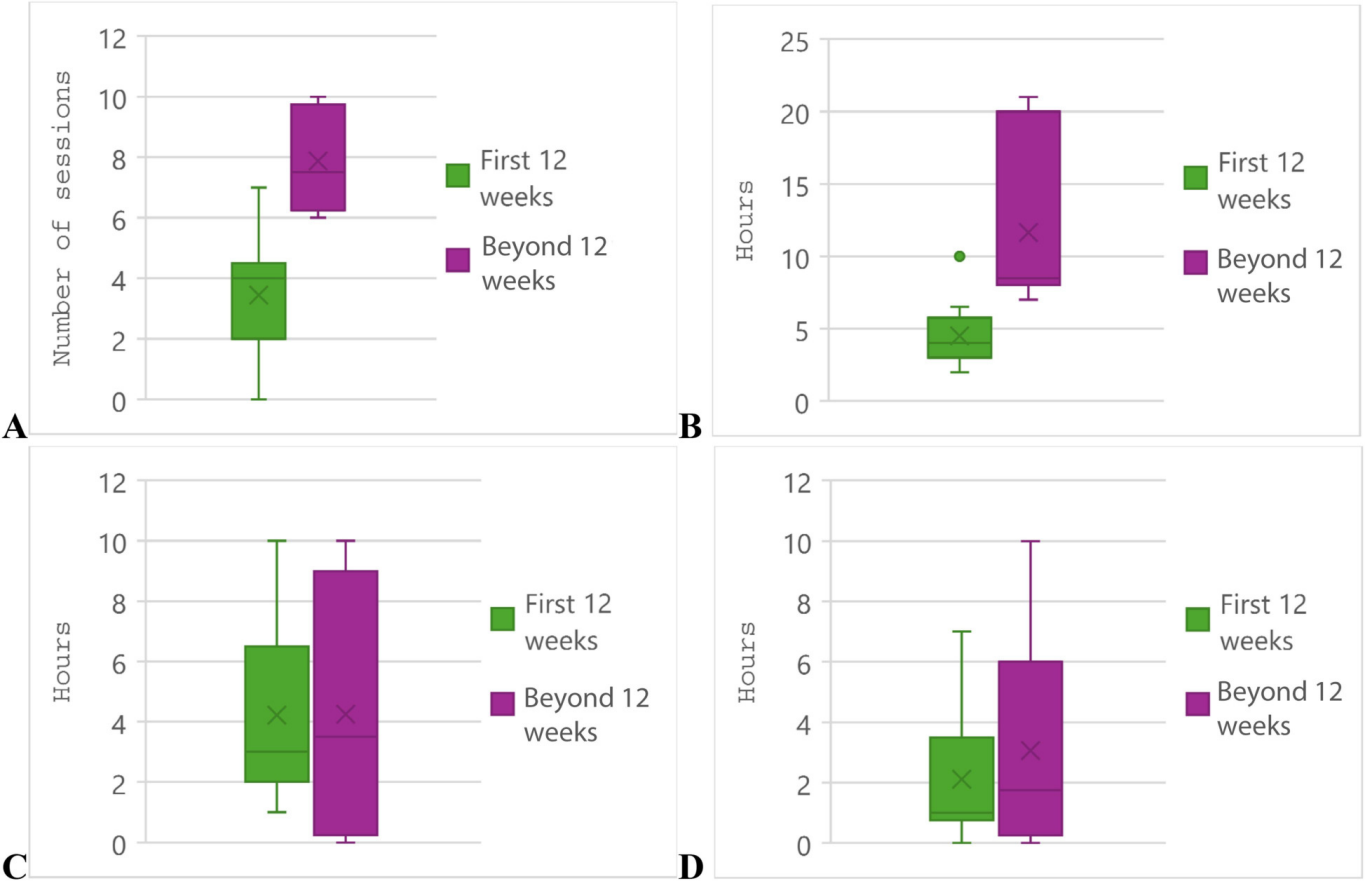
Postpartum training volumes of participants (*N* = 9). First postpartum training session represents *t* = 0. **(A)** Total number of training sessions per week. **(B)** Total hours of training per week. **(C)** Hours of cross-training per week. **(D)** Hours of strength training per week. The “x” denotes the mean and the solid line through the box denotes the median. The whiskers denote the first and third quartiles, and individual data points denote outliers.

While the weekly number of training sessions increased considerably after the first twelve weeks of postpartum training, this represented 2.5 fewer sessions than pre-pregnancy (Figure 4). The median number of weekly sessions postpartum was 7.5 compared to 10 sessions pre-pregnancy. This was also observed in the total hours of training, with a decrease from 12 h to 8.5 h weekly. Athletes slightly increased the weekly hours (3–3.5) of cross training past twelve weeks, despite many not cross-training prior to pregnancy. Hours of strength training increased after twelve weeks of training from a median of one to 1.75 h per week.

Eight of the nine athletes who resumed training postpartum reported to have achieved their pre-pregnancy training level. Of these eight athletes, six (75%) required over 12 weeks to reach their pre-pregnancy training level after more than 12 weeks of training. However, two (25%) athletes achieved this in 10–12 weeks.

Four (44%) athletes referred to postpartum guidelines when returning to training. Two athletes followed recommendations from peer-reviewed journals (17, 19), as well as a published set of physiotherapist-developed guidelines (32). Another athlete followed a walk-run program from a fellow athlete-mother, as well as referring to other connections in the sporting community for strengthening exercises. One athlete followed the general recommendation of starting with gentle movement and gradually reintroducing high intensity exercise. Three of the four athletes who followed postpartum training guidelines felt that they were appropriate for their level of sport. The athlete who did not feel they were suitable described that there was too much emphasis in guidelines, particularly on social media, on the timeline of returning to exercise at six weeks postpartum. This athlete did not feel ready to resume training at this recommended timeline.

Two thirds (*n* = 6) of participants experienced some type of negative training implication during their postpartum return. The most common implications were decreased muscle strength and prolonged recovery time, both experienced by four athletes. One athlete mentioned that their core strength was particularly affected. Other experienced implications were decreased endurance (*n* = 3), decreased training response (*n* = 1), decreased concentration (*n* = 2) and decreased coordination (*n* = 1).

#### 3.6.3 Training advice

Athletes had mixed opinions on their overall satisfaction with the training advice they received during pregnancy and the postpartum period. Of the seven athletes that trained during pregnancy, four (57%) reported being either somewhat (*n* = 2) or extremely (*n* = 2) dissatisfied with training advice received both during pregnancy and the postpartum period. One (14%) athlete described being somewhat satisfied and two (29%) felt neither satisfied nor dissatisfied. Participants were slightly more satisfied with the advice received during the postpartum period compared to during pregnancy; however, still four (57%) athletes were either somewhat (*n* = 3) or extremely (*n* = 1) dissatisfied. Two (29%) athletes reported being somewhat satisfied and one (14%) was neither satisfied nor dissatisfied.

Participants were asked the impact of training advice on confidence in their ability to return to sport postpartum. Athletes were equally distributed between a somewhat negative (*n* = 3, 30%) and somewhat positive (*n* = 3, 30%) impact, and four (40%) athletes described the impact as neutral. Athletes were also asked their opinion on the impact of developing structured postpartum training plans. All athletes felt that it would have a positive impact, with four (40%) ranking it as somewhat positive and six (60%) as extremely positive.

### 3.7 Pyschological impacts

There were no psychological implications reported among athletes during pregnancy, or any symptoms of postpartum depression. Two athletes reported experiencing postpartum anxiety, of which one also experienced irritability during the postpartum period.

For most women, pregnancy and the postpartum period had a positive impact on their mental health. Six out of 10 (60%) athletes described pregnancy as somewhat or extremely positive, and this increased to seven (70%) athletes during the postpartum period. Three (30%) women described pregnancy as having a somewhat negative impact on their mental health; the impact improved during the postpartum period among two of the women and remained the same for the third. One and two athletes described pregnancy and the postpartum period as having neither a positive nor negative impact, respectively.

We asked participants their level of agreement with several statements on emotions associated with balancing motherhood and elite sport (Table 3). Six participants reported somewhat agreeing with feeling guilty pursuing their sport while pregnant, with the other four somewhat or strongly disagreeing. The sense of guilt once their child was born appeared to be somewhat strong with four participants agreeing and three strongly agreeing with feeling guilty pursuing sport while raising their child(ren). There was no uniform opinion on the level of confidence that a career in sport was compatible with motherhood, as levels ranged from “Somewhat disagree” to “Strongly agree.” All but one athlete strongly agreed with the importance of serving as a positive role model in sport for their child(ren).

**Table 3:** Levels of agreement among 10 participants with statements concerning emotions associated with balancing motherhood and elite sport [*n*/*N* (%)].

Statement	Strongly disagree	Somewhat disagree	Neither agree nor disagree	Somewhat agree	Strongly agree
I felt guilty pursuing sport while pregnant	2/10 (20%)	2/10 (20%)	0/10 (0%)	6/10 (60%)	0/10 (0%)
I felt guilty pursuing sport while raising my child(ren)	0/10 (0%)	3/10 (30%)	0/10 (0%)	4/10 (40%)	3/10 (30%)
I felt confident that a career in sport was compatible with motherhood	0/10 (0%)	2/10 (20%)	1/10 (10%)	4/10 (40%)	3/10 (30%)
Being a positive role model for my child(ren) was very important to my postpartum athletic pursuit	0/10 (0%)	0/10 (0%)	1/10 (10%)	0/10 (0%)	9/10 (90%)

### 3.8 Financial support and sport policies

Half of the sample (*n* = 5, 50%) reported to be receiving athletic funding prior to becoming pregnant. All but two (80%) participants reported to have some form of occupation outside of sport, either full time (i.e., >37.5 h per week) or part time (i.e., <37.5 h per week). One athlete reported partaking in full-time studies in addition to having a part-time occupation.

Only one athlete (Athletics) reported having a specific section dedicated to pregnancy in their funding contract. Upon disclosing pregnancy, three athletes reported to have experienced a reduction in funding. Two athletes reported receiving financial support from their sport to go towards childcare. One funded Athletics athlete was given a timeframe to return to sport after childbirth by their sponsor, which they reported was six months. Four athletes reported to have received some form of accommodation for their child when they were training and competing, including having a private hotel room for competitions and allowing the child's grandparents to accompany them at training camp. When asked whether they would use childcare accommodations if offered by their sport, all athletes said yes.

In general, the status of financial support and policies for pregnant and postpartum athletes has been met with some degree of dissatisfaction among survey participants. Three athletes stated that the current support and policies are not satisfactory, and four stated that they have improved as a result of athletes within their sport appealing for change. Two athletes felt that the policies have improved over time. Only one athlete (Basketball) felt that the status of support and policies have always been satisfactory.

When asked how much time should be allowed for parental leave among athletes, eight of the ten (80%) athletes indicated at least one year. Of these eight, two suggested 18 months and two suggested up to two years. Two (20%) athletes suggested individualized timelines rather than a set timeline.

**Figure 5:**
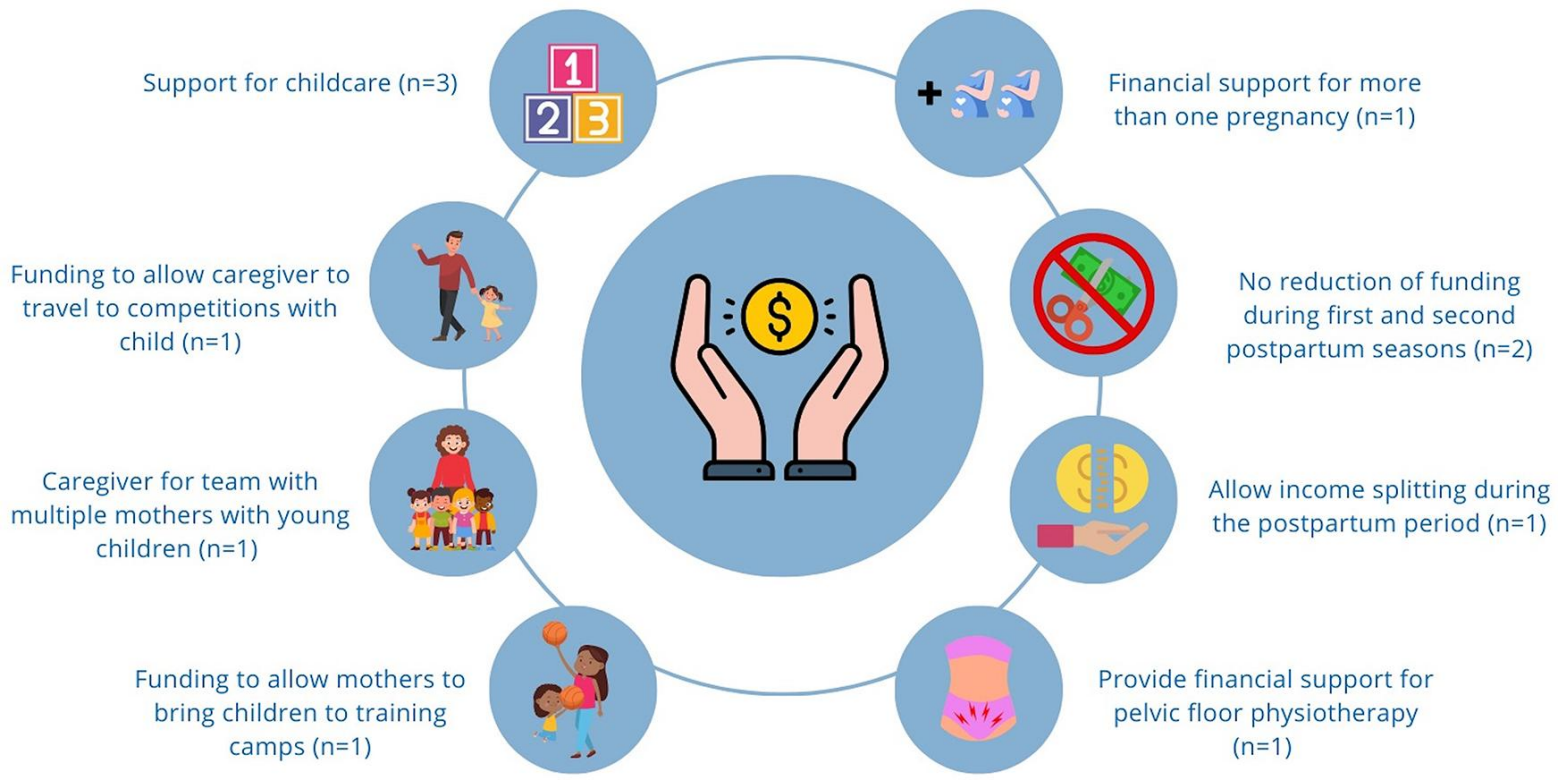
Suggestions among seven participants for improving financial support for pregnant and postpartum athletes.

Participants were given the chance to provide any suggestions to improve financial support for pregnant and postpartum athletes. A reoccurring suggestion was to have a source of funding to specifically go towards childcare, as well as financial support that would allow a caregiver to travel with the athlete and child during competitions and training camps. Two athletes suggested the prevention of funding reductions during the first two sporting seasons postpartum, and one suggested the allowance of income splitting during the postpartum season. Other suggestions for financial support included support for multiple pregnancies as well as a budget for pelvic floor physiotherapy. Figure 5 provides a visual representation to illustrate the variety of suggestions.

### 3.9 Social support

When training or competing, athletes most commonly (*n* = 7, 70%) turned to their partners to look after their children. However, some athletes had a family member (*n* = 2, 20%), or a nanny or babysitter (*n* = 1) serve as the main caregiver when they were occupied with sport. Several athletes (*n* = 6, 60%) described finding an individual to care for their child while they were training or competing as a significant challenge. All athletes agreed that the emotional support of their partner, family members or friends was critical to their postpartum return to sport and performance.

Overall, participants appeared to have a difficult experience seeking healthcare advice while pregnant and postpartum. Most (*n* = 8, 80%) disagreeing that medical staff were knowledgeable in managing this athlete population, and the remaining two (20%) athletes neither agreed nor disagreed. The experience with knowledgeable coaching staff was better, but still most (*n* = 6, 60%) disagreed that their coaching staff provided helpful information for pregnant and postpartum athletes. Only one athlete strongly agreed that their coaching staff were knowledgeable in managing pregnant and postpartum athletes.

### 3.10 Sociocultural influences

The majority of women (*n* = 9, 90%) disagreed that the best suited role for women is to be mothers; the other athlete (*n* = 1, 10%) neither agreed nor disagreed. However, when asked their opinion on the role of parenthood in athletic success, there was less uniformity in the responses. Three (30%) athletes agreed, 3 (30%) disagreed, and the remainder (40%) were neutral that parenthood is the key who wholeness and success as an athlete.

When asked how they were perceived by the public as pregnant athletes, participants were fairly evenly split between positive and negative perceptions, with five (50%) athletes feeling being perceived positively, four (40%) negatively and the remaining athlete feeling neutral. However; the perception of participants as athlete-mothers was generally more positive; seven (70%) athletes felt they were perceived positively, one (10%) neutrally and only two (20%) negatively.

### 3.11 Postpartum performance

Of the nine athletes who resumed training after childbirth, seven (78%) returned to competition. The two athletes who did not described still intending to return but were still in postpartum training at the time of survey completion. The median time to first competition was eight months postpartum (range 4–11).

More athletes experienced improved performance (*n* = 4, 57%) upon their postpartum return than worse performance (*n* = 2, 29%). Of the five athletes who saw improved or equal performance, all of which were Athletics athletes, four required over 12 months to achieve this level while one athlete required less than three months.

Participants were asked to compare the number of competitions in their first postpartum season to their pre-pregnancy amount. The relative amount varied considerably. One athlete competed in less than 10% as many competitions, while three athletes competed more than ever postpartum. The other two athletes ranged from 41% to 60%. In subsequent seasons, all but one athlete were participating in 81 to over 100% of their number of pre-pregnancy competitions. This one athlete reported to be competing at 21%–30% in subsequent seasons.

### 3.12 Subsequent pregnancies

Five athletes had a second child, and four of the five became pregnant during their competitive careers. One athlete gave birth to a third child during their competitive career. All pregnancies were planned.

More pregnancy complications occurred during athletes' second pregnancies. One athlete experienced nausea/vomiting, unexplained vaginal bleeding, nutrient deficiency, weakened immune system, stress or urge incontinence, and pelvic/uterine prolapse. The other athlete experienced gestational hypertension. No participants experienced complications during childbirth. Postpartum complications were similar as described in Section 3.3.

Injuries during pregnancy and the postpartum period appeared to be as common as in prior pregnancies. One athlete experienced Achilles and quadriceps tendinopathy postpartum, which they described as a recurring issue prior to their second pregnancy.

One athlete had an increase in funding prior to becoming pregnant with their second child but then had their funding cut by more than half in their first season following pregnancy.

Athletes who trained while pregnant reported training further into subsequent pregnancies compared to their first. Compared to their first pregnancy, most reported increasing their volume, intensity, and amounts of strength training and cross training.

Athletes typically resumed training earlier and increase the frequency and intensity of their training compared to their postpartum training after their first pregnancy. Only one athlete reported not resuming training after their second pregnancy; however, they described transitioning to training for a new sport.

Compared to their postpartum performance after their first birth, all athletes that returned to competitive sport were able to return to at least the same performance level. Most athletes even saw improved performance in subsequent pregnancies.

When asked to provide any additional insight on their first pregnancy compared to subsequent pregnancies, some athletes had polarizing experiences. One athlete described the consequences of a poorly timed second pregnancy; they competed three weeks postpartum and as a result experienced significant abdominal separation. The second athlete described having a much easier return to sport during their second pregnancy as a result of consistent spin-bike training and weightlifting throughout pregnancy which prevented significant muscle loss. The athlete with three children described having fewer opportunities to compete when compared to previous seasons as a result of raising young children.

### 3.13 Athlete reflections

Of the seven athletes who have to date returned to competitive sport after childbirth, five mentioned that finding childcare options was a significant barrier to their postpartum return. Three athletes identified breastfeeding as a challenge due to the time and energy required. Two athletes indicated that injuries posed a challenge to their return to performance, and one athlete described the time required for their body to recover as a barrier. Lastly, one athlete described ensuring proper nutrition as well as the lack of a structured return to sport training as making their return more difficult.

All but one athlete described the importance of their spouses and/or family in their postpartum return, both in terms of emotional encouragement and for helping with childcare. One athlete identified strength training as the largest contributor to their postpartum return.

## 4 Discussion

Elite athlete-mothers are a relatively new population in sports medicine research due to social and sport culture norms that have often viewed motherhood as being an all-consuming identity (1, 25, 33, 34). Therefore, exploring the experiences of pregnancy and return to sport and performance postpartum in elite athletes is critical to advance knowledge.

Athletes in the current study and previous studies have demonstrated that mothers can be successful athletes (9, 20–22, 24, 25, 28, 29, 35, 36). Findings of our study suggest that, despite the barriers that still exist, several athlete-mothers of our sample were able to return to performance postpartum and even achieve lifetime bests in their sport. However, many identify gaps in support. We speculate that if support for these athletes is improved, the population of athletes extending their careers into motherhood and excelling may increase.

In general, the findings in our study reflect those of previous publications of elite athlete-mothers (2, 24, 25, 27, 35, 37–40). In addition, new findings revealed in this study may suggest recent changes to policy and culture, or a different athlete population than previously studied. Differences in geography and sport disciplines may account for variance in several factors, such availability of funding, training/education of coaches and medical staff, and cultural norms towards women and mothers in sport. This variance highlights the need for ongoing research, as priorities may change as the population of pregnant and postpartum athletes grows.

### 4.1 Training

A previous study exploring the experiences of elite athletes through pregnancy noted that participants commonly expressed concern for the impact of their training on fetal health (40). Many athletes in the present study continued sport-specific training through pregnancy without reporting having safety concerns for their baby. This may suggest that despite the ongoing lack of training guidance, knowledge has improved that elite athletes are generally able to safely train during pregnancy.

Pregnant and postpartum athletes of previous studies have described a lack of trust in existing training guidelines, with emphasis generally on the need for a structured return to sport framework (2, 9, 15, 40). Interestingly, athletes in our sample expressed more dissatisfaction with available guidelines for training during pregnancy rather than guidelines postpartum. In fact, one athlete reported not training while pregnant as a result of a lack of training advice. Another athlete described that their training through their second pregnancy contributed to their easier return to performance compared to their first pregnancy, highlighting the need for more evidence-based training recommendations, particularly during pregnancy.

A qualitative study exploring the experience of coaches and health care providers on working with pregnant and postpartum athletes revealed that the experts in this sample recommend a milestone-based approach to return to sport, with emphasis on restoring pelvic floor strength (38). Only one athlete in our sample expressed the desire for more access to pelvic floor health care. The return to sport sources to which athletes in our study made reference were primarily timeline-based (19, 32).

Previous literature has found that medical clearance to resume physical activity is generally given at six weeks postpartum (2, 9, 15, 41, 42). However, a scoping review of 33 postpartum return to activity and return sport recommendations revealed that there is no formal criteria used when assessing readiness (15). Most athletes in our sample who resumed training did so by five weeks postpartum. Whether they received medical clearance was not explored, but it is possible that some athletes used this 6-week benchmark as their personal criteria to resume training. One athlete expressed feeling pressure to resume training by six weeks as a result of the strong emphasis on this timeline in the information resources to which they were referring. Athletes who experienced complicated deliveries, maternal or fetal health complications, or psychological implications postpartum may not feel ready to return to sport by this six-week recommendation. In fact, several reviews suggest that athletes with Caesarean deliveries require additional recovery time before resuming exercise (15, 43, 44). Despite this recommendation, the two athletes who underwent Caesarean deliveries resumed training at five and six weeks postpartum. In addition to increased education on pregnancy and the postpartum period among sport support staff, return to sport recommendations should further emphasize an individualized rather than a generalized timeline to minimize risk of complications or pressure felt by athletes.

Participants were asked to provide details on the guidelines which they referred to for training during pregnancy and the postpartum period. The responses revealed that athletes were generally not obtaining training recommendations from evidence-based guidelines. During pregnancy, athletes sought advice from physicians, who may not have been formally trained in sports medicine. Only one athlete referred to a true postpartum return to sport training guideline (32); the others referring to advice from a fellow athlete, online recommendations and training patterns from postpartum athletes in a peer-reviewed journal. Further inspection of the referred guideline revealed that most recommendations were based on expert opinions and lacked high-level certainty of evidence (32). This may suggest a lack of awareness of or access to existing evidence-based recommendations for training during pregnancy and the postpartum period (13, 43–46).

### 4.2 Injury/pain

Eight out of 10 athletes in our study suffered an injury or musculoskeletal pain during the postpartum period. One athlete even reported being unable to resume postpartum training to date as a result of injury that was neglected during pregnancy. Differences in types and locations of injuries were noted from previous work. Several studies have identified the sacrum as a common area for stress fractures among postpartum athletes (13, 18, 21). Only one athlete in our sample experienced bone stress reactions (in the fibula and patella). The most common region of postpartum injury or musculoskeletal pain appeared to be the Achilles tendon in our study. Regardless of the differences, postpartum injuries continue to be of high prevalence and warrant further exploration (2, 19, 22, 47). It is possible that these injuries are a consequence of resuming training without being fully recovered from childbirth, as suggested by the lack of evidence-based advice to which our sample was referring. This further highlights the need for improving the delivery of safe return to sport recommendations to athletes. Additionally, injury prevention methods for postpartum athletes that are different from the general athlete population may promote a more successful return to sport and performance.

Based on the finding that athletes in our sample may be unaware of evidence-based training guidelines, combined with the high prevalence of postpartum injury, future research could explore perceptions of training during pregnancy and the postpartum period. Insight on how and where athletes are seeking training recommendations may reveal required improvements for accessibility of information—either via publishable guidelines or training of coaching and medical staff. Whether athletes are seeking medical clearance or referring to any criteria for returning to training postpartum may reveal additional information on the nature of training advice received, such as timeline- or milestone-based. This in turn may highlight modifications in recommendations and promote an individualistic return to training.

### 4.3 Mental health

Athletes in the current study generally described pregnancy and the postpartum period as having a positive impact on their mental health and confidence in sport participation. Another recent study, on the contrary, described elite athletes experiencing mental and emotional difficulties during their postpartum return to performance, with one athlete experiencing symptoms of postpartum depression (2). It is possible that funded athletes in our study faced fewer external pressures to make a swift postpartum return to performance due to improved sponsor policies, and/or had better access to mental health resources. Differences in cultures between sports may also contribute to the contrast in psychological impacts among studies, highlighting the need for knowledge on a broader athlete population.

### 4.4 Financial support

Athletes in a previous qualitative study described funding as being an important consideration when planning a pregnancy (40). Only five athletes in the study indicated they received athletic funding, and pregnancy planning was primarily described as being influenced by competition schedules rather than athletic funding. Although several described feeling pressure to conceive and give birth in time to return to performance for an upcoming competitive season, only one athlete felt pressure from their sponsor to make a rapid return. Athletes in the aforementioned study emphasized the importance of financial support from their sport in their ability to return to performance after childbirth (40). While athletes in our study generally agreed that funding could be improved for pregnant and postpartum athletes, this was not described as a critical factor in their postpartum return. This may be reflective of the experiences of a slightly different athlete population—high-level athletes who are not sponsored or receive significant funding. It is also possible that financial support for athletes has improved since the publication of the previous study, such that the desire to quickly return to sport and performance postpartum is a result of personal motivation, and not due to a need for financial support.

Future research could further explore differences in experiences between funded and non-funded athletes. While changes to sport policies regarding pregnancy and parental leave would likely benefit funded athletes, identifying other areas of need may drive positive changes that could help a broader population of athletes.

### 4.5 Subsequent pregnancies

Although only a small number of participants reported subsequent pregnancies during their athletic career, to our knowledge this is the first study to describe experiences of return to sport and performance across pregnancies. While pregnancy, childbirth and postpartum experiences were similar to prior pregnancies, athletes commonly reported training further into pregnancy, as well as increasing the duration and intensity of their sessions when compared to their first pregnancy. Postpartum training was also typically resumed earlier compared to previous pregnancies. It is possible that training in subsequent pregnancies was modified on the basis of personal experience.

### 4.6 Contextualization of key findings

The experiences described by athlete-mothers suggest that there may be a sense of urgency to return to competitive sport after childbirth. This begins with the pressure to conceive within a certain timeframe to not miss competition and continues with a return to training just weeks after birth. Several athletes reported to still be breastfeeding when they resumed competing, facing energy challenges and sleep disturbances. Consequences of this rush back into sport and performance may manifest as the postpartum injuries and complications related to pelvic floor function that were commonly described.

Future research could aim to explore the sources of the perceived need for a rapid postpartum return to sport and performance. If sport policies have expected timelines for athlete-mothers to resume training and competing, the establishment of such timelines could be explored, and whether they have referred to scientific data on postpartum recovery. How athletes are setting goals for return to sport and performance timelines, and if criteria for readiness are being considered may provide insight on whether a safe return to sport is being encouraged.

In general, there is dissatisfaction with training advice for pregnancy and the postpartum period among athletes. Athletes in our sample appeared to be unaware of evidence-based guidelines and were dissatisfied with advice received from coaching and medical staff. Exploring how athletes are seeking training advice for pregnancy and the postpartum period may identify challenges in current access of evidence-based information for this athlete population. Similarly, knowledge of coaches and medical staff on sport participation through pregnancy and the postpartum period may highlight areas of need for improved education.

## 5 Limitations

The sampling method applied in our study can result in selection bias. Mutual connections may limit the sample to athletes from similar sporting disciplines, or those with similar pregnancy and postpartum experiences. Likewise, volunteer bias may exist, as it is possible that the sample only consists of athletes with very positive or negative return to sport and performance experiences. The lack of formal scientific validation of the questionnaire may reduce the internal validity of the findings. Due to the small sample size, the study was limited to descriptive findings and is not generalizable to athletes outside of the study sample. The generalizability is further limited due to most athletes participating in Athletics, as well as all participants being white, Canadian, cis-gender women who were all legally married and not separated. Information bias, due to self-report based on recall, was also a potential issue in this study, posing a threat on internal validity.

## 6 Conclusion

This study provided preliminary insight on the experiences of a small sample of Canadian elite athletes who attempted to return to sport and performance after childbirth. Despite experiencing challenges and uncertainty, most athletes were able to return to performance after childbirth, with several even achieving improved performance postpartum. Several athletes described dissatisfaction with training advice received during pregnancy and the postpartum period, and none referred to evidence-based guidelines. Combined with the high prevalence of injuries, this highlights the need for future initiatives to improve the delivery of safe training and return to sport. This study also provides new insight on experiences of new athlete-mothers, non-funded athletes and athletes who return to sport and performance after multiple pregnancies. With some observations varying from previous findings, this study demonstrates the need for additional research on pregnant and postpartum athletes to work towards the identification of the most critical support mechanisms. It is the hope that the observations of athlete experiences in this sample contribute to the growing body of literature with the aims of making sport excellence and enjoyment within reach for more of an athlete-mother's career.

## Data Availability

The raw data supporting the conclusions of this article will be made available by the authors, without undue reservation.
