# Supplementary material for: Exploring the postpartum return to sport and performance in Canadian elite athletes
Source: Front Sports Act Living. 2025 Oct 7;7:1665212. doi: 10.3389/fspor.2025.1665212 (PMC12537676; doi:10.3389/fspor.2025.1665212)
Supplement: Supplementary file 1 [file Datasheet1.docx]

Supplementary material: Study questionnaire

Return to Performance Postpartum

Start of Block: Eligibility Screening

Q1.1 Are you 18 years of age or older?

- Yes (1)
- No (2)

Q1.2 Have you met the requirements to compete at the national level in your sport (either pre-pregnancy or postpartum)?

- Yes (1)
- No (2)

Q1.3 Is your sport funded by Sport Canada?

- Yes (1)
- No (2)

Q1.4 Have you become pregnant during your athletic career and delivered a child?

- Yes (1)
- No (2)

Q1.5 Did you attempt to return to competitive sport after childbirth?

- Yes (1)
- No (2)

| Page Break |  |
| --- | --- |

Display This Question:

If Are you 18 years of age or older? = Yes

And Have you become pregnant during your athletic career and delivered a child? = Yes

And Did you attempt to return to competitive sport after childbirth? = Yes

And Have you met the requirements to compete at the national level in your sport (either pre-pregnanc... = Yes

And Is your sport funded by Sport Canada? = Yes

Q1.6 Please review the Letter of Information below prior to proceeding with consent to participate in the study.

 **LETTER OF INFORMATION AND CONSENT FORM**

 **Project Title:**
 Determinants of postpartum return to performance in Canadian elite athletes

 **Principal Investigator:**
 Dr. Jane Thornton Fowler Kennedy Sport Medicine Clinic, Western University 3M Centre 1151 Richmond Street London, ON N6A 3K7 519-661-3011 | jane.thornton@schulich.uwo.ca

 **Study Coordinator:**
 Chloe Hewitt, MSc candidate Department of Epidemiology and Biostatistics, Western University chewit3@uwo.ca

 **Co-Investigator:**
 Dr. Karen Campbell Department of Epidemiology and Biostatistics, Western University Centre for Public Health and Family Medicine 1465 Richmond Street London, ON N6G 2M1 519-661-2162 | mcampbel@uwo.ca

 **Introduction and Study Background**
 You are reading this because you expressed interest in participating in a masters survey-based study at Western University.

Athlete-mothers are an increasing sub-population among elite athletes. Until recently, motherhood and a career in sport were considered incompatible. As a result, there is little research and guidance on supporting these athletes towards a healthy and successful return to performance postpartum. This study will explore the most significant factors associated with successfully returning to competition after childbirth, and compare characteristics and experiences of athletes who returned to competition postpartum versus those who were unable to return. Results gained from this study will identify further areas of research and possible improvements to sport policies to promote health and longevity in female sport participation.

You are being asked to complete an online questionnaire. This letter contains information to help you decide whether or not to participate in this research study. It is important for you to understand why the study is being conducted and what it will involve. Please take the time to read this carefully and feel free to contact the study team to ask questions if anything is unclear.

 **Who can participate?**
You are invited to participate if you: have become pregnant during your sporting career, have successfully delivered a child and attempted to return to competitive sport after childbirth. You must at least meet the qualification criteria to compete at the national level in a sport that is funded by Sport Canada. You may verify whether your sport is eligible by referring to the following link: https://www.canada.ca/en/canadian-heritage/services/sport-organizations/national.html. You must also be English speaking and have access to a mobile device or laptop or desktop computer with a working keyboard and internet connection. If you retired prior to becoming pregnant or did not attempt to return to competition after childbirth, are an athlete in a sport not funded by Sport Canada, are non-English speaking or do not have access to a mobile device or computer or internet, you will be excluded.

Due to the difficulty in identifying potential participants, we are asking those who complete the survey to distribute information about our study to athletes who may be eligible and interested in participating.

 **How can I participate?**
 If you meet the eligibility criteria above, you can consent to participate in an online questionnaire. The questionnaire consists of a minimum of ten sections: demographics and sport background, pregnancy planning, pregnancy experiences, breastfeeding, pain and injuries, psychological impacts, financial support and sport policies, social support, and sociocultural influences, and training patterns. If applicable, further sections on postpartum performance and subsequent children during your sporting career will appear, depending on your responses in previous sections. The online questionnaire takes about 45 minutes to complete, but the length depends on which questions are applicable to you based on whether you resumed training/competing after childbirth and how many children you had during your athletic career.

 **What are the potential benefits?**
 You may not experience any benefit from participation. However, this research is relevant to the pregnant and postpartum athletic community, and athletes planning to start a family. With the results of the study, we plan to create a publication to present to National Sporting Organizations (NSOs). This publication will provide recommendations for policy changes to better support pregnant and postpartum athletes as they continue their career into motherhood. We will also contribute to a growing body of research which will further our understanding about factors contributing to a healthy and successful return to performance postpartum to support the next generation of athlete-mothers.

 **What are the risks to participating?**
 The survey will be completed on Qualtrics, a third-party platform. There is always a potential risk of data breach with any survey conducted online. We plan to uphold research integrity to the highest standard to prevent this from occurring. You will not be asked to identify yourself on the survey at any point. Study data will be downloaded and deleted from the Qualtrics server, and stored on our secure Western University network server at the end of the study. The Qualtrics server is located in Ireland. You may review the Qualtrics privacy statement at https://www.qualtrics.com/privacy-statement/ .

 It is possible that you may have questions or concerns about your health after completing this survey. We encourage you to bring these concerns to your doctor as needed.

 **How will we keep your information safe during and after the study?**
 Data from the study will be password-protected and stored on encrypted servers. When communicating study results in publications or presentations, only aggregate data will be presented and no identifying information will be mentioned. Data will be stored for seven years following completion of the study, when it will then be permanently deleted. Our study was reviewed by the Health Sciences Research Ethics Board (REB) at Western University. The REB has access to study information for monitoring, legal, and/or regulatory purposes only.

 **Can I withdraw from the study?**
 Participation in this study is voluntary. You may refuse to answer any survey question if you do not feel comfortable doing so. If you exit the questionnaire before clicking “submit” on the final page, no data will be collected. After submission of the survey, should you decide, you would like to remove your data, you can contact the study researchers to locate your data (based off time of submission, age range and/or other answers) to remove your entire submission.

 **What are the alternatives to being in the study?**
 An alternative to the procedures described above is to not participate in the study and continue on just as you do now.

 **What are the costs and or reimbursement involved in the study?**
 There are no costs for you to partake in the study. You will not be compensated for your participation in this research.

 **Who can I contact about the study?**
 If you have questions or concerns, please contact the Study Coordinator, Chloe Hewitt at chewit3@uwo.ca. If you have any questions about concerns that may be raised by participating in the study or questions that may be raised by being a research participant, please contact our Principal Investigator, Dr. Jane Thornton at 519-661-3011 or jane.thornton@schulich.uwo.ca. If you have any questions about your rights as a research participant or the conduct of this study, you may contact The Office of Human Research Ethics at 519-661-3036 or ethics@uwo.ca.

 **Consent**
 If you choose to participate, consent will be implied upon starting the survey. Electronic consent will be obtained in the survey if you pass the eligibility screening questions.

 Thank you.

 Sincerely,
 Chloe Hewitt, MSc candidate

- I consent to participate (1)
- I do not consent to participate (2)

Skip To: End of Survey If Please review the Letter of Information below prior to proceeding with consent to participate in... = I do not consent to participate

Display This Question:

If Are you 18 years of age or older? = No

Or Have you become pregnant during your athletic career and delivered a child? = No

Or Did you attempt to return to competitive sport after childbirth? = No

Or Have you met the requirements to compete at the national level in your sport (either pre-pregnanc... = No

Or Is your sport funded by Sport Canada? = No

|  |
| --- |

Q1.7 We thank you for your interest; however, you are ineligible to participate in this study.

End of Block: Eligibility Screening

Start of Block: Demographics and Sport Background

| 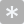 |
| --- |

Q2.1 What is your current age?

________________________________________________________________

Q2.2 Which best describes your current gender identity?

- Male (1)
- Female (2)
- Indigenous cultural gender minority identity or other cultural gender minority identity (e.g., Two-Spirit) (3)
- Self Identity (e.g., gender fluid, non-binary) (4) __________________________________________________
- Unsure (5)
- Prefer not to say (6)

Q2.3 What best describes the racial or cultural group(s) to which you belong? Please select all that apply.

- White (1)
- South Asian (e.g., Indian, Pakistani, Sri Lankan) (2)
- Chinese (3)
- Black (4)
- Filipino (5)
- Latin American (6)
- Arab (7)
- Southeast Asian (e.g., Vietnamese, Cambodian, Malaysian, Laotian) (8)
- West Asian (e.g., Iranian, Afghan) (9)
- Korean (10)
- Japanese (11)
- Pacific Islander (12)
- Indigenous (13)
- Self Identity - please specify (14) __________________________________________________
- ⊗Prefer not to say (15)
- ⊗Unknown (16)

Q2.4 What is your marital status?

- Never legally married (1)
- Legally married (and not separated) (2)
- Separated, but still legally married (3)
- Common-law (4)
- Divorced (5)
- Widowed (6)

| Page Break |  |
| --- | --- |

Q2.5 What is your primary sport and event (if applicable)?

________________________________________________________________

Q2.6 Which province or territory would you compete under as an athlete?

▼ Alberta (1) ... Yukon (13)

| 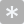 |
| --- |

Q2.7 How many years total have you participated in your sport?

________________________________________________________________

| 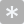 |
| --- |

Q2.8 How many years have you participated in your sport at the elite level (i.e., training to meet the qualification criteria to compete at the national level)?

________________________________________________________________

Q2.9 Have you competed as a member of a senior national team?

- Yes (1)
- No (2)

Q2.10 What is your highest level of competition?

- National competition (1)
- International competition (2)
- Olympic Games or World Championships (3)

Q2.11 Are you a paralympic athlete?

- No (1)
- Yes (please indicate your classification) (2) __________________________________________________

Q2.12 Have you retired from elite sport?

- No (2)
- Yes (please indicate the year you retired) (1) __________________________________________________

End of Block: Demographics and Sport Background

Start of Block: Pregnancy Planning

Q3.1 Was your first pregnancy planned?

- Yes (1)
- No (2)

Skip To: End of Block If Was your first pregnancy planned? = No

Q3.2 If you timed your pregnancy with respect to your competition schedule, please describe how you did so.

________________________________________________________________

Q3.3 How long did it take to conceive?

▼ < 1 month (1) ... > 12 months (14)

Q3.4 Was there pressure to conceive within a certain timeframe?

- No (2)
- Yes (please specify external pressure) (1) __________________________________________________

End of Block: Pregnancy Planning

Start of Block: Pregnancy experiences

Q4.1 How many children have you given birth to?

- 1 (1)
- 2 (2)
- 3 (3)
- 4 (4)
- 5 (5)
- 6 (6)
- 7 (7)
- 8 (8)

| Page Break |  |
| --- | --- |

Q4.2 Please answer the following questions as they pertain to your **first** pregnancy. Questions regarding any subsequent pregnancies will be addressed in later sections.

| Page Break |  |
| --- | --- |

| 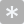 |
| --- |

Q4.3 Please enter your age at the birth of your first child.

________________________________________________________________

Q4.4 What was the delivery method of your first child?

- Vaginal (1)
- Caesarean (2)
- Assisted vaginal (e.g. vacuum, forceps) (3)

Skip To: Q4.6 If What was the delivery method of your first child? = Caesarean

Q4.5 Did you have an episiotomy (*i.e.,*surgical incision of perineum to enlarge vaginal opening) with your first pregnancy?

- Yes (1)
- No (2)

Q4.6 Did you experience any physical complications during your first pregnancy?

- Yes (1)
- No (2)

Skip To: Q4.10 If Did you experience any physical complications during your first pregnancy? = No

Q4.7 Please indicate all of the pregnancy complications that you experienced during your first pregnancy.

- Nausea/vomiting (1)
- Gestational diabetes (2)
- Preeclampsia and/or gestational hypertension (pregnancy-induced high blood pressure) (3)
- Premature labour (4)
- Preterm delivery (5)
- Unexplained vaginal bleeding (6)
- Placenta previa after 28 weeks (abnormal implantation of the placenta at or near the internal opening of the uterine cervix) (7)
- Incompetent cervix (weak cervical tissue contributing to premature birth) (8)
- Type 1 diabetes (9)
- Type 2 diabetes (10)
- Thyroid disease (11)
- Nutrient deficiency (12)
- Anemia (22)
- Excess weight gain (13)
- Intrauterine growth restriction (estimated fetal weight less than the 10th percentile for gestational age) (14)
- Weakened immune system (15)
- Stress/urge incontinence (involuntary leakage of urine from the bladder) (16)
- Fecal incontinence (involuntary leakage of stool from rectum) (18)
- Antenatal sepsis (organ dysfunction caused by an infection during pregnancy) (20)
- Pelvic/uterine prolapse (falling of pelvic floor muscles from their usual position) (21)
- Multiple births (i.e., twins, etc.) - please indicate how many (19) __________________________________________________
- Other - please specify (17) __________________________________________________

Q4.8 Did you experience any childbirth complications during your first pregnancy?

- Yes (1)
- No (2)

Skip To: Q4.10 If Did you experience any childbirth complications during your first pregnancy? = No

Q4.9 Please indicate all of the childbirth complications you experienced during your first pregnancy.

- Prolonged labour (4)
- Shoulder dystocia (one of both of infant's shoulders stuck during vaginal delivery) (5)
- Placenta previa (abnormal implantation of the placenta at or near the internal opening of the uterine cervix) (6)
- Excessive bleeding (2)
- Uterine rupture (tearing of uterus) (7)
- Perineal tear (vaginal tearing) - please specify grade (1) __________________________________________________
- Other - please specify (3) __________________________________________________

Q4.10 Did you experience any physical complications postpartum with your first child?

- Yes (1)
- No (2)

Skip To: End of Block If Did you experience any physical complications postpartum with your first child? = No

Q4.11 Please indicate all of the complications that you experienced postpartum with your first child.

- Stress/urge incontinence (involuntary leakage of urine from the bladder) (1)
- Fecal incontinence (involuntary leakage of stool from rectum) (16)
- Rectus abdominis diastasis (abdominal muscle separation) (3)
- Vaginal heaviness/pressure (6)
- Excess weight loss (7)
- Weakened immune system (8)
- Irregular menstrual cycle (9)
- Gastrointestinal issues (10)
- Low bone mineral density (11)
- Nutrient deficiency (12)
- Postnatal sepsis (organ dysfunction caused by an infection in the postpartum period) (17)
- Pelvic/uterine prolapse (falling of pelvic floor muscles from their usual position) (18)
- Other - please specify (13) __________________________________________________

End of Block: Pregnancy experiences

Start of Block: Breastfeeding

Q5.1 Did you breastfeed your first child? (Can include both nursing and pumping, exclusive breastfeeding and supplementing with formula).

- Yes (1)
- No (2)

Skip To: End of Block If Did you breastfeed your first child? (Can include both nursing and pumping, exclusive breastfeedi... = No

Q5.2 How long did you breastfeed for?

▼ < 1 month (14) ... > 12 months (13)

| Page Break |  |
| --- | --- |

Q5.3 Did you train while breastfeeding your first child?

- Yes (1)
- No (2)

Q5.4 Please rate the level of difficulty you experienced incorporating breastfeeding into your training schedule.

- Extremely difficult (13)
- Somewhat difficult (14)
- Neither easy nor difficult (15)
- Somewhat easy (16)
- Extremely easy (17)
- N/A (20)

Q5.5 Did breastfeeding affect your training and/or competition?

- No (1)
- Yes - please describe how (2) __________________________________________________
- N/A (3)

Q5.6 Were you still breastfeeding when you first returned to competition?

- Yes (1)
- No (2)
- N/A (3)

Skip To: End of Block If Were you still breastfeeding when you first returned to competition? = No

Q5.7 Did you ever experience any issues bringing your breastfeeding child to your competitions?

- No (2)
- Yes - please describe (1) __________________________________________________

End of Block: Breastfeeding

Start of Block: Injuries/pain

Q6.1 Did you suffer from any injuries or musculoskeletal pain while pregnant?

- No (2)
- Yes - please specify location and type (e.g. joint pain, sprain, strain, tear, tendinopathy, stress fracture, fracture, etc.) (1) __________________________________________________

Q6.2 Did you experience any injuries or musculoskeletal pain during the postpartum period?

- No (2)
- Yes - please specify location and type (e.g. joint pain, sprain, strain, tear, tendinopathy, stress fracture, fracture, etc.) (1) __________________________________________________

Display This Question:

If Did you suffer from any injuries or musculoskeletal pain while pregnant? = Yes - please specify location and type (e.g. joint pain, sprain, strain, tear, tendinopathy, stress fracture, fracture, etc.)

Or Did you experience any injuries or musculoskeletal pain during the postpartum period? = Yes - please specify location and type (e.g. joint pain, sprain, strain, tear, tendinopathy, stress fracture, fracture, etc.)

Q6.3 Were any of the aforementioned injuries or pain a recurring issue prior to pregnancy?

- Yes (1)
- No (2)

End of Block: Injuries/pain

Start of Block: Psychological Impacts

Q7.1 Please indicate the effect of the following on your mental health:

|  | Extremely negative (1) | Somewhat negative (2) | Neither positive nor negative (3) | Somewhat positive (4) | Extremely positive (5) |
| --- | --- | --- | --- | --- | --- |
| Pregnancy (1) |  |  |  |  |  |
| Postpartum period (2) |  |  |  |  |  |

Q7.2 Did you experience any psychological implications during pregnancy?

- Yes (1)
- No (2)

Skip To: Q7.4 If Did you experience any psychological implications during pregnancy? = No

Q7.3 Please indicate any of the following psychological implications you experienced during pregnancy.

- Anxiety (1)
- Depression (2)
- Fear of movement (3)
- Irritability (4)
- Body image concerns (5)
- Disordered eating (6)
- Eating disorder (8)
- Other (please specify) (7) __________________________________________________

Q7.4 Did you experience any symptoms of postpartum depression?

- Yes, diagnosed (1)
- Suspected, but not diagnosed (2)
- No (3)
- Unsure (4)

Q7.5 Did you experience any other psychological implications during the postpartum period?

- Yes (1)
- No (2)

Skip To: Q7.7 If Did you experience any other psychological implications during the postpartum period? = No

Q7.6 Please indicate any of the following psychological implications you experienced during the postpartum period.

- Anxiety (1)
- Fear of movement (2)
- Irritability (3)
- Body image concerns (4)
- Disordered eating (5)
- Eating disorder (7)
- Other (please specify) (6) __________________________________________________

Q7.7 How much do you agree with the following statements?

|  | Strongly disagree (1) | Somewhat disagree (2) | Neither agree nor disagree (3) | Somewhat agree (4) | Strongly agree (5) |
| --- | --- | --- | --- | --- | --- |
| I felt guilty pursuing sport while pregnant (4) |  |  |  |  |  |
| I felt guilty pursuing sport while raising my child(ren) (1) |  |  |  |  |  |
| I felt confident that a career in sport was compatible with motherhood (2) |  |  |  |  |  |
| Being a positive role model for my child(ren) was very important to my postpartum athletic pursuit (3) |  |  |  |  |  |

End of Block: Psychological Impacts

Start of Block: Financial Support and Sport Policies

Q8.1 Were you receiving athlete funding prior to pregnancy?

- Yes (1)
- No (2)

Q8.2 Did your funding status change upon becoming pregnant?

- No (1)
- Yes, reduction of funding (2)
- Yes, increase in funding (3)
- Yes, lost funding (4)

Q8.3 Do you have another occupation apart from sport? Select all that apply.

- No (1)
- Yes, I work part time (less than 37.5 hours/week) (2)
- Yes, I work full time (37.5 hours/week or more) (3)
- Yes, I am a part-time student (4)
- Yes, I am a full-time student (5)

Q8.4 Were you provided with any financial support for childcare through your sport?

- Yes - please indicate who provided the support (1) __________________________________________________
- No (2)
- Unsure (3)
- N/A (4)

Q8.5 Did your sponsor or sport organization provide a timeframe in which you were to return to competition?

- Yes (please indicate how many months after childbirth) (1) __________________________________________________
- No (2)
- N/A (3)

Q8.6 Did your funding contracts have a section designated to policies surrounding pregnancy?

- Yes (1)
- No (2)
- Unsure (3)
- N/A (4)

Q8.7 Did your sport organization provide access to accommodation for your child(ren) while training or competing?

- Yes - please describe accommodation (1) __________________________________________________
- No (2)
- N/A (3)

Q8.8 If offered, would you use the accommodation for your child(ren) provided by your sport organization?

- Yes (1)
- No (2)

Q8.9 Please select the statement that best describes the status of financial support for pregnant and postpartum athletes within your sport.

- The support and policies for pregnant and postpartum athletes have always been satisfactory within my sport. (1)
- The support and policies for pregnant and postpartum athletes have improved as a result of fellow athletes appealing for change. (2)
- The support and policies for pregnant and postpartum athletes have improved over time. (3)
- The support and policies for pregnant and postpartum athletes are not satisfactory within my sport. (4)

Q8.10 In your opinion, how much time should be allowed for parental leave among athletes?

________________________________________________________________

Q8.11 If you have any suggestions to improve financial support for pregnant and postpartum athletes, please provide them in the space below.

________________________________________________________________

End of Block: Financial Support and Sport Policies

Start of Block: Social Support

Q9.1 Who most regularly looked after your child when you were unavailable due to training or competition?

- Partner/spouse (1)
- Parent (2)
- Other family member (3)
- Friend (4)
- Nanny/babysitter (5)
- Other (please specify) (6) __________________________________________________
- N/A (7)

Q9.2 How much do you agree with the following statements?

|  | Strongly disagree (1) | Somewhat disagree (2) | Neither agree nor disagree (3) | Somewhat agree (4) | Strongly agree (5) |
| --- | --- | --- | --- | --- | --- |
| The emotional support of a partner/family member/friend contributed significantly to my ability to return to training postpartum. (1) |  |  |  |  |  |
| Finding someone to care for my child while I was training/competing was a considerable challenge. (2) |  |  |  |  |  |

Q9.3 How much do you agree with the following statements?

|  | Strongly diasagree (1) | Somewhat disagree (2) | Neither agree nor disagree (3) | Somewhat agree (4) | Strongly agree (5) |
| --- | --- | --- | --- | --- | --- |
| Medical staff were knowledgeable in treating pregnant and postpartum athletes (1) |  |  |  |  |  |
| Coaching staff were knowledgeable in providing training advice to pregnant and postpartum athletes (2) |  |  |  |  |  |

End of Block: Social Support

Start of Block: Sociocultural Influences

Q10.1 How much do you agree with the following statements?

|  | Strongly disagree (1) | Somewhat disagree (2) | Neither agree nor disagree (3) | Somewhat agree (4) | Strongly agree (5) |
| --- | --- | --- | --- | --- | --- |
| Women are best suited to take on the single identity of being a mother as opposed to having multiple identities (1) |  |  |  |  |  |
| Parenthood is the key to wholeness and success as an athlete (2) |  |  |  |  |  |

Q10.2 How do you feel you were perceived:

|  | Extremely negatively (1) | Somewhat negatively (2) | Neither positively nor negatively (3) | Somewhat positively (4) | Extremely positively (5) | Not applicable (6) |
| --- | --- | --- | --- | --- | --- | --- |
| As a pregnant athlete? (1) |  |  |  |  |  |  |
| As an athlete-mother? (2) |  |  |  |  |  |  |

End of Block: Sociocultural Influences

Start of Block: Training Patterns

Q11.1 Did you train during your pregnancy?

- Yes (1)
- No - please explain why not (2) __________________________________________________

Skip To: Q11.9 If Did you train during your pregnancy? = No - please explain why not

Q11.2 Please select all of the trimesters in which you trained.

- First trimester (1)
- Second trimester (2)
- Third trimester (3)

Display This Question:

If Please select all of the trimesters in which you trained. != First trimester

Or Please select all of the trimesters in which you trained. != Second trimester

Or Please select all of the trimesters in which you trained. != Third trimester

Q11.3 Please indicate why you did not train during a particular trimester.

________________________________________________________________

| 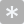 |
| --- |

Q11.4 Up until how many weeks did you train into your pregnancy?

________________________________________________________________

| Page Break |  |
| --- | --- |

| 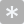 |
| --- |

Q11.5 Please complete the following values pertaining to your average weekly training over the course of your pregnancy (if you did not train during a particular trimester, please enter 0).

|  | Pre-pregnacy (1) | First trimester (2) | Second trimester (3) | Third trimester (4) |
| --- | --- | --- | --- | --- |
| Number of training sessions (1) |  |  |  |  |
| Total hours of training (2) |  |  |  |  |
| Number of cross training sessions (4) |  |  |  |  |
| Number of strength training sessions (3) |  |  |  |  |

Q11.6 Please indicate how your training intensity over the course of your pregnancy compared to your pre-pregnancy training intensity.

|  | Significantly decreased (1) | Slightly decreased (2) | Remained the same (3) | Slightly increased (4) | Significantly increased (5) | N/A - did not train (8) |
| --- | --- | --- | --- | --- | --- | --- |
| First trimester (1) |  |  |  |  |  |  |
| Second trimester (2) |  |  |  |  |  |  |
| Third trimester (3) |  |  |  |  |  |  |

| Page Break |  |
| --- | --- |

Q11.7 Did you refer to any guidelines for training recommendations throughout pregnancy?

- No (2)
- Yes - please indicate which guideline(s) you used (1) __________________________________________________

Skip To: Q11.9 If Did you refer to any guidelines for training recommendations throughout pregnancy? = No

Q11.8 Did you feel that the guidelines were applicable to your level of sport?

- Yes (1)
- No - please explain why not (2) __________________________________________________

| Page Break |  |
| --- | --- |

Q11.9 Did you resume training after childbirth?

- Yes (1)
- No - please explain why not (2) __________________________________________________

Skip To: Q11.19 If Did you resume training after childbirth? = No - please explain why not

| 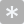 |
| --- |

Q11.10 How many weeks postpartum was your first training session?

________________________________________________________________

Q11.11 What was the nature of your first postpartum training session?

- Sport-specific training (1)
- Cross training (2)

| Page Break |  |
| --- | --- |

| 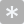 |
| --- |

Q11.12 Please complete the following values pertaining to your average weekly training throughout the course of the postpartum period (with your first training session after childbirth as the starting point).

|  | First 12 weeks (1) | Beyond 12 weeks (2) |
| --- | --- | --- |
| Number of training sessions (1) |  |  |
| Total hours of training (2) |  |  |
| Hours of cross training (4) |  |  |
| Hours of strength training (11) |  |  |

Q11.13 Once you resumed training, after how many weeks of postpartum training was your first high-intensity session?

- <6 weeks (1)
- 6-8 weeks (2)
- 8-10 weeks (3)
- 10-12 weeks (4)
- >12 weeks (5)

Q11.14 Once you resumed training, after how many weeks were you training at your pre-pregnancy level?

- <6 weeks (1)
- 6-8 weeks (2)
- 8-10 weeks (3)
- 10-12 weeks (4)
- >12 weeks (5)
- I never reached my pre-pregnancy training level (6)

| Page Break |  |
| --- | --- |

Q11.15 Did you refer to any guidelines for postpartum training recommendations?

- No (2)
- Yes - please indicate which guideline(s) you used (1) __________________________________________________

Skip To: Q11.17 If Did you refer to any guidelines for postpartum training recommendations? = No

Q11.16 Did you feel that the guidelines were applicable to your level of sport?

- Yes (1)
- No - please explain why not (2) __________________________________________________

| Page Break |  |
| --- | --- |

Q11.17 Did you experience any negative implications when training and/or competing postpartum?

- Yes (1)
- No (2)

Skip To: Q11.19 If Did you experience any negative implications when training and/or competing postpartum? = No

Q11.18 Please select all of the negative implications you experienced while training and/or competing postpartum.

- Decreased muscle strength (1)
- Decreased endurance (2)
- Decreased training response (3)
- Decreased coordination (4)
- Decreased concentration (5)
- Impaired judgement (6)
- Prolonged recovery time (7)
- Other - please specify (8) __________________________________________________

| Page Break |  |
| --- | --- |

Q11.19 Please indicate your level of satisfaction with the training advice you received: 

|  | Extremely dissatisfied (1) | Somewhat dissatisfied (2) | Neither satisfied nor dissatisfied (3) | Somewhat satisfied (4) | Extremely satisfied (5) | Not applicable (6) |
| --- | --- | --- | --- | --- | --- | --- |
| During pregnancy (1) |  |  |  |  |  |  |
| During the postpartum period (2) |  |  |  |  |  |  |

Q11.20 How did your level of satisfaction with training advice affect your confidence in returning to sport postpartum?

- Extremely negative (1)
- Somewhat negative (2)
- Neither positive nor negative (3)
- Somewhat positive (4)
- Extremely positive (5)

Q11.21 In your opinion, what impact would the development of a structured postpartum training plan have on athletes?

- Extremely negative (1)
- Somewhat negative (2)
- Neither positive nor negative (3)
- Somewhat positive (4)
- Extremely positive (5)

End of Block: Training Patterns

Start of Block: Postpartum Performance

Q12.1 Did you compete in your sport after giving birth?

- Yes (1)
- No - please explain why not (2) __________________________________________________

Skip To: End of Block If Did you compete in your sport after giving birth? = No - please explain why not

| Page Break |  |
| --- | --- |

| 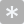 |
| --- |

Q12.2 How many months after giving birth was your first competition?

________________________________________________________________

Q12.3 How does your postpartum performance level compare to your pre-pregnancy level?

- Significantly worse (1)
- Somewhat worse (2)
- About the same (3)
- Somewhat improved (4)
- Significantly improved (5)

Display This Question:

If How does your postpartum performance level compare to your pre-pregnancy level? = About the same

Or How does your postpartum performance level compare to your pre-pregnancy level? = Somewhat improved

Or How does your postpartum performance level compare to your pre-pregnancy level? = Significantly improved

Q12.4 Once you resumed competing, how long did it take you to be performing at your pre-pregnancy level (or better)?

- < 3 months (1)
- 3-5 months (2)
- 6-8 months (3)
- 9-11 months (5)
- 12+ months (6)

Q12.5 During your first season after giving birth, what was the relative amount of competitions you participated in compared to pre-pregnancy?

▼ < 10% (1) ... > 100% (11)

Q12.6 During your subsequent seasons after giving birth, what was the relative amount of competitions you participated in compared to pre-pregnancy?

▼ < 10% (1) ... Not applicable (12)

End of Block: Postpartum Performance

Start of Block: Reflections

Q13.1 In your opinion, what was the biggest barrier in returning to performance postpartum?

________________________________________________________________

Q13.2 In your opinion, what was the biggest aid in returning to performance postpartum?

________________________________________________________________

End of Block: Reflections

Start of Block: Subsequent Children - Second Child

Q14.1 When did you become pregnant with your second child?

- During competitive career (1)
- After competitive career (2)

Skip To: End of Survey If When did you become pregnant with your second child? = After competitive career

| 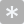 |
| --- |

Q14.2 Please enter your age at the birth of your second child.

________________________________________________________________

Q14.3 Was your second pregnancy planned?

- Yes (1)
- No (2)

Q14.4 What was the delivery method of your second child?

- Vaginal (1)
- Caesarean (2)
- Assisted vaginal (e.g. vacuum, forceps) (3)

Skip To: Q14.6 If What was the delivery method of your second child? = Caesarean

Q14.5 Did you have an episiotomy (*i.e.,* surgical incision of perineum to enlarge vaginal opening) with your second pregnancy?

- Yes (1)
- No (2)

Q14.6 Did you experience any physical complications during your second pregnancy?

- Yes (1)
- No (2)

Skip To: Q14.10 If Did you experience any physical complications during your second pregnancy? = No

Q14.7 Please indicate all of the complications that you experienced during your second pregnancy.

- Nausea/vomiting (1)
- Gestational diabetes (2)
- Preeclampsia and/or gestational hypertension (pregnancy-induced high blood pressure) (3)
- Premature labour (4)
- Preterm delivery (5)
- Unexplained vaginal bleeding (6)
- Placenta previa after 28 weeks (abnormal implantation of the placenta at or near the internal opening of the uterine cervix) (7)
- Incompetent cervix (weak cervical tissue contributing to premature birth) (8)
- Type 1 diabetes (9)
- Type 2 diabetes (10)
- Thyroid disease (11)
- Nutrient deficiency (12)
- Anemia (22)
- Excess weight gain (13)
- Intrauterine growth restriction (estimated fetal weight less than the 10th percentile for gestational age) (14)
- Weakened immune system (15)
- Stress/urge incontinence (involuntary leakage of urine from the bladder) (16)
- Fecal incontinence (involuntary leakage of stool from rectum) (18)
- Pelvic/uterine prolapse (falling of pelvic floor muscles from their usual position) (21)
- Antenatal sepsis (organ dysfunction caused by an infection during pregnancy) (20)
- Multiple births (i.e., twins, etc.) - please indicate how many (19) __________________________________________________
- Other - please specify (17) __________________________________________________

Q14.8 Did you experience any childbirth complications during your second pregnancy?

- Yes (1)
- No (2)

Skip To: Q14.10 If Did you experience any childbirth complications during your second pregnancy? = No

Q14.9 Please indicate all of the childbirth complications you experienced during your second pregnancy.

- Prolonged labour (4)
- Shoulder dystocia (one of both of infant's shoulders stuck during vaginal delivery) (5)
- Placenta previa previa (abnormal implantation of the placenta at or near the internal opening of the uterine cervix) (6)
- Excessive bleeding (2)
- Uterine rupture (tearing of uterus) (7)
- Perineal tear (vaginal tearing) - please specify grade (1) __________________________________________________
- Other - please specify (3) __________________________________________________

Q14.10 Did you experience any physical complications postpartum with your second child?

- Yes (1)
- No (2)

Skip To: Q14.12 If Did you experience any physical complications postpartum with your second child? = No

Q14.11 Please indicate all of the postpartum complications that you experienced with your second child.

- Stress/urge incontinence (involuntary leakage of urine from the bladder) (1)
- Fecal incontinence (involuntary leakage of stool from rectum) (14)
- Rectus abdominis diastasis (abdominal muscle separation) (3)
- Vaginal heaviness/pressure (6)
- Excess weight loss (7)
- Weakened immune system (8)
- Irregular menstrual cycle (9)
- Gastrointestinal issues (10)
- Low bone mineral density (11)
- Nutrient deficiency (12)
- Postnatal sepsis (organ dysfunction caused by an infection in the postpartum period) (15)
- Pelvic/uterine prolapse (falling of pelvic floor muscles from their usual position) (16)
- Other - please specify (13) __________________________________________________

| Page Break |  |
| --- | --- |

Q14.12 Did you suffer from any injuries or musculoskeletal pain during your second pregnancy?

- No (2)
- Yes - please specify location and type (e.g. joint pain, sprain, strain, tear, tendinopathy, stress fracture, fracture, etc.) (1) __________________________________________________

Q14.13 Did you experience any postpartum injuries or musculoskeletal pain with your second child?

- No (2)
- Yes - please specify location and type (e.g. joint pain, sprain, strain, tear, tendinopathy, stress fracture, fracture, etc.) (1) __________________________________________________

Display This Question:

If Did you suffer from any injuries or musculoskeletal pain while pregnant? = Yes - please specify location and type (e.g. joint pain, sprain, strain, tear, tendinopathy, stress fracture, fracture, etc.)

Or Did you experience any injuries or musculoskeletal pain during the postpartum period? = Yes - please specify location and type (e.g. joint pain, sprain, strain, tear, tendinopathy, stress fracture, fracture, etc.)

Q14.14 Were any of the aforementioned injuries or pain a recurring issue prior to your second pregnancy?

- Yes (1)
- No (2)

| Page Break |  |
| --- | --- |

Q14.15 Did you experience any psychological implications during your second pregnancy?

- Yes (1)
- No (2)

Skip To: Q14.17 If Did you experience any psychological implications during your second pregnancy? = No

Q14.16 Please indicate any of the following psychological implications you experienced during your second pregnancy.

- Anxiety (1)
- Depression (2)
- Fear of movement (3)
- Irritability (4)
- Body image concerns (5)
- Disordered eating (6)
- Eating disorder (8)
- Other - please specify (7) __________________________________________________

Q14.17 Did you experience any psychological implications postpartum with your second child?

- Yes (1)
- No (2)

Skip To: Q14.19 If Did you experience any psychological implications postpartum with your second child? = No

Q14.18 Please indicate any of the following psychological implications you experienced during the postpartum period with your second child.

- Anxiety (1)
- Depression (2)
- Fear of movement (3)
- Irritability (4)
- Body image concerns (5)
- Disordered eating (6)
- Eating disorder (8)
- Other - please specify (7) __________________________________________________

| Page Break |  |
| --- | --- |

Q14.19 Did you breastfeed your second child? (Can include both nursing and pumping, exclusive breastfeeding and supplementing with formula).

- Yes (1)
- No (2)

Skip To: Q14.23 If Did you breastfeed your second child? (Can include both nursing and pumping, exclusive breastfeed... = No

Q14.20 How long did you breastfeed for?

▼ < 1 month (1) ... > 12 months (5)

Q14.21 Did you train while breastfeeding your second child?

- Yes (1)
- No (2)

Skip To: Q14.23 If Did you train while breastfeeding your second child? = No

Q14.22 Did breastfeeding affect your training and/or competition?

- No (1)
- Yes - please describe how (2) __________________________________________________

| Page Break |  |
| --- | --- |

Q14.23 Did your funding status change between your first and second pregnancy?

- Yes (1)
- No (2)

Skip To: Q14.25 If Did your funding status change between your first and second pregnancy? = No

Q14.24 How did your funding status change?

- Increase in funding (1)
- Decrease in funding (2)
- Lost funding completely (3)

Q14.25 If you have any additional comments regarding funding during your second pregnancy, please add them in the space below.

________________________________________________________________

| Page Break |  |
| --- | --- |

Q14.26 Did you train during your second pregnancy?

- Yes (1)
- No - please explain why not (2) __________________________________________________

Skip To: Q14.29 If Did you train during your second pregnancy? = No - please explain why not

Q14.27 Please select the appropriate responses comparing training during your second pregnancy to your first pregnancy.

|  | Decreased (1) | Remained the same (2) | Increased (3) |
| --- | --- | --- | --- |
| Number of training sessions per week (1) |  |  |  |
| Average training volume (2) |  |  |  |
| Average training intensity (3) |  |  |  |
| Amount of strength training (4) |  |  |  |
| Amount of cross training (5) |  |  |  |

Q14.28 How did the duration of your training change in your second pregnancy?

- I trained further into my pregnancy (1)
- I trained shorter into my pregnancy (2)
- It did not change (3)

| Page Break |  |
| --- | --- |

Q14.29 Did you resume training after your second pregnancy?

- Yes (1)
- No - please explain why not (2) __________________________________________________

Skip To: Q14.38 If Did you resume training after your second pregnancy? = No - please explain why not

Q14.30 Please select the appropriate responses comparing postpartum training following second pregnancy to your first pregnancy.

|  | Decreased (1) | Remained the same (2) | Increased (3) |
| --- | --- | --- | --- |
| Number of training sessions per week (1) |  |  |  |
| Average training volume (2) |  |  |  |
| Average training intensity (3) |  |  |  |
| Amount of strength training (4) |  |  |  |
| Amount of cross training (5) |  |  |  |

Q14.31 How did the timing of your return to training after your second pregnancy compare to your first pregnancy?

- I returned to training earlier in the postpartum period (1)
- I returned to training later in the postpartum period (2)
- It did not change (3)

Q14.32 Did you experience any negative implications when training and/or competing postpartum with your second child?

- Yes (1)
- No (2)

Q14.33 Please select all of the negative implications you experienced while training and/or competing postpartum with your second child.

- Decreased muscle strength (1)
- Decreased endurance (2)
- Decreased training response (3)
- Decreased coordination (4)
- Decreased concentration (5)
- Impaired judgement (6)
- Prolonged recovery time (7)
- Other - please specify (8) __________________________________________________

| Page Break |  |
| --- | --- |

Q14.34 Did you return to competitive sport after your second pregnancy?

- Yes (1)
- No - please explain why not (2) __________________________________________________

Skip To: Q14.38 If Did you return to competitive sport after your second pregnancy? = No - please explain why not

Q14.35 How did the timing of your first postpartum following your second pregnancy competition compare to your previous pregnancy?

- I returned earlier (1)
- I returned later (2)
- I returned around the same time (3)

Q14.36 How did your athletic performance after your second pregnancy compare to your previous pregnancy?

- Improved (1)
- Remained the same (2)
- Worsened (3)

Q14.37 In the seasons following your second pregnancy, how did the amount of competitions you participated in compare to after your previous pregnancy?

- I competed more (1)
- I competed less (2)
- I competed around the same amount (3)

| Page Break |  |
| --- | --- |

Q14.38 If you have any additional comments regarding your experience with your second pregnancy compared to your first pregnancy, please add them in the space below.

________________________________________________________________

End of Block: Subsequent Children - Second Child

Start of Block: Subsequent Children - Third Child

Q15.1 When did you become pregnant with your third child?

- During competitive career (1)
- After competitive career (2)

Skip To: End of Survey If When did you become pregnant with your third child? = After competitive career

Q15.2 Was your third pregnancy planned?

- Yes (1)
- No (2)

| 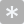 |
| --- |

Q15.3 Please enter your age at the birth of your third child.

________________________________________________________________

Q15.4 What was the delivery method of your third child?

- Vaginal (1)
- Caesarean (2)
- Assisted vaginal (e.g. vacuum, forceps) (3)

Skip To: Q15.6 If What was the delivery method of your third child? = Caesarean

Q15.5 Did you have an episiotomy (*i.e.*, surgical incision of perineum to enlarge vaginal opening) with your third pregnancy?

- Yes (1)
- No (2)

Q15.6 Did you experience any physical complications during your third pregnancy?

- Yes (1)
- No (2)

Skip To: Q15.10 If Did you experience any physical complications during your third pregnancy? = No

Q15.7 Please indicate all of the physical complications that you experienced during your third pregnancy.

- Nausea/vomiting (1)
- Gestational diabetes (2)
- Preeclampsia and/or gestational hypertension (pregnancy-induced high blood pressure) (3)
- Premature labour (4)
- Preterm delivery (5)
- Unexplained vaginal bleeding (6)
- Placenta previa after 28 weeks (abnormal implantation of the placenta at or near the internal opening of the uterine cervix (7)
- Incompetent cervix (weak cervical tissue contributing to premature birth) (8)
- Type 1 diabetes (9)
- Type 2 diabetes (10)
- Thyroid disease (11)
- Nutrient deficiency (12)
- Anemia (22)
- Excess weight gain (13)
- Intrauterine growth restriction (estimated fetal weight less than the 10th percentile for gestational age) (14)
- Weakened immune system (15)
- Stress/urge incontinence (involuntary leakage of urine from the bladder) (16)
- Fecal incontinence (involuntary leakage of stool from rectum) (19)
- Antenatal sepsis (organ dysfunction caused by an infection during pregnancy) (20)
- Pelvic/uterine prolapse (falling of pelvic floor muscles from their usual position) (21)
- Multiple births (i.e., twins, etc.) - please indicate how many (18) __________________________________________________
- Other - please specify (17) __________________________________________________

Q15.8 Did you experience any childbirth complications during your third pregnancy?

- Yes (1)
- No (2)

Skip To: Q15.10 If Did you experience any childbirth complications during your third pregnancy? = No

Q15.9 Please indicate all of the childbirth complications you experienced during your third pregnancy.

- Prolonged labour (4)
- Shoulder dystocia (one of both of infant's shoulders stuck during vaginal delivery) (5)
- Placenta previa (abnormal implantation of the placenta at or near the internal opening of the uterine cervix) (6)
- Excessive bleeding (2)
- Uterine rupture (tearing of uterus) (7)
- Perineal tear (vaginal tearing) - please specify grade (1) __________________________________________________
- Other - please specify (3) __________________________________________________

Q15.10 Did you experience any physical complications during the postpartum period with your third child?

- Yes (1)
- No (2)

Skip To: Q15.12 If Did you experience any physical complications during the postpartum period with your third child? = No

Q15.11 Please indicate all of the complications that you experienced postpartum with your third child.

- Stress/urge incontinence (involuntary leakage of urine from the bladder) (1)
- Fecal incontinence (involuntary leakage of stool from rectum) (16)
- Rectus abdominus diastasis (abdominal muscle separation) (3)
- Vaginal heaviness/pressure (6)
- Excess weight loss (7)
- Weakened immune system (8)
- Irregular menstrual cycle (9)
- Gastrointestinal issues (10)
- Low bone mineral density (11)
- Nutrient deficiency (12)
- Postnatal sepsis (organ dysfunction caused by an infection in the postpartum period) (17)
- Pelvic/uterine prolapse (falling of pelvic floor muscles from their usual position) (18)
- Other - please specify (13) __________________________________________________

| Page Break |  |
| --- | --- |

Q15.12 Did you suffer from any injuries or musculoskeletal pain during your third pregnancy?

- No (2)
- Yes - please specify location and type (e.g. joint pain, sprain, strain, tear, tendinopathy, stress fracture, fracture, etc.) (1) __________________________________________________

Q15.13 Did you experience any injuries or musculoskeletal pain during the postpartum period with your third child?

- No (2)
- Yes - please specify location and type (e.g. joint pain, sprain, strain, tear, tendinopathy, stress fracture, fracture, etc.) (1) __________________________________________________

Display This Question:

If Did you suffer from any injuries or musculoskeletal pain while pregnant? = Yes - please specify location and type (e.g. joint pain, sprain, strain, tear, tendinopathy, stress fracture, fracture, etc.)

Or Did you experience any injuries or musculoskeletal pain during the postpartum period? = Yes - please specify location and type (e.g. joint pain, sprain, strain, tear, tendinopathy, stress fracture, fracture, etc.)

Q15.14 Were any of the aforementioned injuries or pain a recurring issue prior to your third pregnancy?

- Yes (1)
- No (2)

| Page Break |  |
| --- | --- |

Q15.15 Did you experience any psychological implications during your third pregnancy?

- Yes (1)
- No (2)

Skip To: Q15.17 If Did you experience any psychological implications during your third pregnancy? = No

Q15.16 Please indicate all of the following psychological implications you experienced during your third pregnancy.

- Anxiety (1)
- Depression (2)
- Fear of movement (3)
- Irritability (4)
- Body image concerns (5)
- Disordered eating (8)
- Eating disorder (6)
- Other - please specify (7) __________________________________________________

Q15.17 Did you experience any psychological implications during the postpartum period with your third child?

- Yes (1)
- No (2)

Skip To: Q15.19 If Did you experience any psychological implications during the postpartum period with your third ch... = No

Q15.18 Please indicate any of the following psychological implications you experienced during the postpartum period with your third child.

- Anxiety (1)
- Depression (2)
- Fear of movement (3)
- Irritability (4)
- Body image concerns (5)
- Disordered eating (8)
- Eating disorder (6)
- Other - please specify (7) __________________________________________________

| Page Break |  |
| --- | --- |

Q15.19 Did you breastfeed your third child? (Can include both nursing and pumping, exclusive breastfeeding and supplementing with formula).

- Yes (1)
- No (2)

Skip To: Q15.23 If Did you breastfeed your third child? (Can include both nursing and pumping, exclusive breastfeedi... = No

Q15.20 How long did you breastfeed for?

- < 1 month (1)
- 1 month (2)
- 2 months (3)
- 3 months (4)
- 4 months (6)
- 5 months (7)
- 6 months (8)
- 7 months (9)
- 8 months (10)
- 9 months (11)
- 10 months (12)
- 11 months (13)
- 12 months (14)
- > 12 months (5)

Q15.21 Did you train while breastfeeding your third child?

- Yes (1)
- No (2)

Skip To: Q15.23 If Did you train while breastfeeding your third child? = No

Q15.22 Did breastfeeding affect your training and/or competition?

- No (1)
- Yes - please describe how (2) __________________________________________________

| Page Break |  |
| --- | --- |

Q15.23 Did your funding status change between your second and third pregnancy?

- Yes (1)
- No (2)

Skip To: Q15.25 If Did your funding status change between your second and third pregnancy? = No

Q15.24 How did your funding status change?

- Increase in funding (1)
- Decrease in funding (2)
- Lost funding completely (3)

Q15.25 If you have any additional comments regarding funding during your third pregnancy, please add them in the space below.

________________________________________________________________

| Page Break |  |
| --- | --- |

Q15.26 Did you train during your third pregnancy?

- Yes (1)
- No - please explain why not (2) __________________________________________________

Skip To: Q15.29 If Did you train during your third pregnancy? = No - please explain why not

Q15.27 Please select the appropriate responses comparing training during your third pregnancy to your previous pregnancies.

|  | Decreased (1) | Remained the same (2) | Increased (3) |
| --- | --- | --- | --- |
| Number of training sessions per week (1) |  |  |  |
| Average training volume (2) |  |  |  |
| Average training intensity (3) |  |  |  |
| Amount of strength training (4) |  |  |  |
| Amount of cross training (5) |  |  |  |

Q15.28 How did the duration of your training change in your third pregnancy?

- I trained further into my pregnancy (1)
- I trained shorter into my pregnancy (2)
- It did not change (3)

| Page Break |  |
| --- | --- |

Q15.29 Did you resume training after your third pregnancy?

- Yes (1)
- No - please explain why not (2) __________________________________________________

Skip To: Q15.38 If Did you resume training after your third pregnancy? = No - please explain why not

Q15.30 Please select the appropriate responses comparing postpartum training following third pregnancy to your previous pregnancies.

|  | Decreased (1) | Remained the same (2) | Increased (3) |
| --- | --- | --- | --- |
| Number of training sessions per week (1) |  |  |  |
| Average training volume (2) |  |  |  |
| Average training intensity (3) |  |  |  |
| Amount of strength training (4) |  |  |  |
| Amount of cross training (5) |  |  |  |

Q15.31 How did the timing of your return to training after your third pregnancy compare to your previous pregnancies?

- I returned to training earlier in the postpartum period (1)
- I returned to training later in the postpartum period (2)
- It did not change (3)

Q15.32 Did you experience any negative implications when training and/or competing postpartum with your third child?

- Yes (1)
- No (2)

Q15.33 Please select all of the negative implications you experienced while training and/or competing postpartum with your third child.

- Decreased muscle strength (1)
- Decreased endurance (2)
- Decreased training response (3)
- Decreased coordination (4)
- Decreased concentration (5)
- Impaired judgement (6)
- Prolonged recovery time (7)
- Other - please specify (8)

| Page Break |  |
| --- | --- |

Q15.34 Did you return to competitive sport after your third pregnancy?

- Yes (1)
- No - please explain why not (2) __________________________________________________

Skip To: Q15.38 If Did you return to competitive sport after your third pregnancy? = No - please explain why not

Q15.35 How did the timing of your first postpartum competition following your third pregnancy compare to your previous pregnancies?

- I returned earlier (1)
- I returned later (2)
- I returned around the same time (3)

Q15.36 How did your athletic performance after your third pregnancy compare to your previous pregnancies?

- Improved (1)
- Remained the same (2)
- Worsened (3)

Q15.37 In the seasons following your third pregnancy, how did the amount of competitions you participated in compare to after your previous pregnancy?

- I competed more (1)
- I competed less (2)
- I competed around the same amount (3)

| Page Break |  |
| --- | --- |

Q15.38 If you have any additional comments regarding your experience with your third pregnancy compared to your previous pregnancies, please add them in the space below.

________________________________________________________________

End of Block: Subsequent Children - Third Child

Start of Block: Future recruitment

Q21.1 Thank you for taking the time to complete our survey. In order to gather as much data as possible on this important topic, we kindly encourage you to share the information about our study with other athletes who may be interested.

End of Block: Future recruitment
